# Characterization of induced cohesin loop extrusion trajectories in living cells

**DOI:** 10.1038/s41588-025-02358-0

**Published:** 2025-10-16

**Authors:** Ruiqi Han, Yike Huang, Michelle J. Robers, Mikhail Magnitov, Iwan Vaandrager, Amin Allahyar, Marjon J. A. M. Verstegen, Kexin Zhang, Elzo de Wit, Wouter de Laat, Peter H. L. Krijger

**Affiliations:** 1 Oncode Institute, Hubrecht Institute-KNAW and University Medical Center Utrecht, Utrecht, the Netherlands; 2 Division of Gene Regulation, The Netherlands Cancer Institute, Amsterdam, the Netherlands; 3 Present Address: Institute of Modern Biology, Nanjing University, Nanjing, China; 4 Present Address: Department of Genetics, University Medical Center Utrecht, Utrecht University, Utrecht, the Netherlands

**Keywords:** Epigenomics, Epigenetics

## Abstract

Cohesin (SMC1–SMC3–RAD21) constantly extrudes DNA loops to organize chromosomes into structural domains, pausing and anchoring at specific DNA-bound CTCF molecules. To study the detailed consequences of cohesin loop extrusion, we developed TArgeted Cohesin Loader (TACL) for controlled pan-cellular activation of chromatin loop formation at defined genomic locations in living cells. With TACL, we show that highly complex looping networks can exist, with extruding cohesin complexes that block each other, drive cohesin queuing and induce loop anchoring at nearly all CTCF-bound sites. TACL loops extend upon acute depletion of STAG2, PDS5A or WAPL. Activated cohesin loop extrusion hinders local gene transcription and can alter chromatin accessibility and H3K27ac distribution. TACL shows that the loading/extrusion complex NIPBL–MAU2 can be transported by cohesin to CTCF sites but, together with SMC1, to enhancers in a RAD21-independent manner. TACL thus enables studying the consequences of activated loop extrusion at defined genomic locations.

## Abstract (web summary)

This study introduces a system called TArgeted Cohesin Loader (TACL) that recruits cohesin complexes at defined genomic regions and induces loop extrusion events in living cells, exploring its impact on chromatin organization and gene expression.

## Main

The evolutionarily conserved cohesin complex is a tripartite, ring-shaped structure consisting of RAD21, SMC1 and SMC3, associated with either STAG1 or STAG2. The complex functions to hold sister chromatids together during mitosis and to shape chromosomes in interphase cells by forming topologically associating domains (TADs)^1–3^. Cohesin establishes TADs presumably by loop extrusion^4–6^ in which the complex is loaded on chromatin and subsequently reels in flanking sequences to build progressively larger DNA loops in an ATP-dependent manner^7–10^. STAG1 and STAG2 have overlapping but non-redundant functions. STAG1-associated cohesin is more stably associated with chromatin and is crucial to establishing the longer chromatin loops between CTCF-associated domain boundaries. STAG2-associated cohesin was reported to be more often found at non-CTCF sites and form intra-TAD loops between enhancers and genes^11–13^. Cohesin cycles between a chromatin-bound and chromatin-extruding state and an unbound state^14,15^. It requires NIPBL and MAU2 for stable chromatin association^16,17^ and loop extrusion processivity^18–21^, while WAPL releases cohesin from chromatin and restricts loop extrusion^22^. When reaching convergently oriented CTCF proteins that demarcate domain boundaries, cohesin is protected against WAPL-mediated release^22^: it stalls and forms temporarily stabilized chromatin loops between opposite domain boundaries^15,23–25^. PDS5 interacts with cohesin and localizes on chromosomes at CTCF-bound loop anchors^3,26,27^. It is thought to compete with NIPBL for binding to cohesin^26,28^ and, similar to CTCF, PDS5 functions to restrict chromatin loop sizes^3,29,30^. How loop extrusion impacts transcription remains unclear. Recent evidence suggests that the transcription and loop extrusion machineries interact when traversing chromatin^31–33^ and that continuous cohesin-mediated loop extrusion is required for the regulation of developmental genes by distal enhancers^14,34–37^. Studying the impact of cohesin loop extrusion activity in vivo remains challenging, however^38^, as individual loop extrusion trajectories are difficult to manipulate in living cells. Furthermore, in vivo studies of cohesin largely rely on (acute) cohesin protein depletion, which leads to widespread changes in chromatin structure and functioning, cell cycle arrest and cell death^1,14,15^. Systems that enable local control of cohesin loop extrusion activity at defined genomic locations in healthy living cells will help to more accurately monitor the direct consequences of altered loop extrusion activity.

## Results

### TACL system

**Fig. 1:**
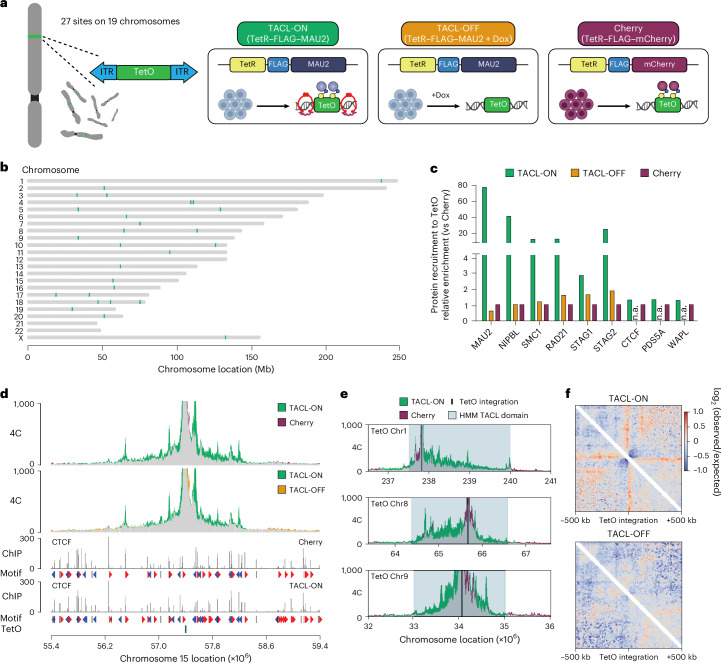
TACL recruits the functional cohesin complex at designated genomic sites. **a**, Schematic representation of the TACL system using an HAP1 cell line with 27 TetO platforms in its genome. TACL-ON (green) and TACL-OFF (orange) are TACL cells expressing TetR–FLAG–MAU2. TACL-OFF cells were treated with doxycycline for 1 h. Cherry HAP1 cells (purple) expressed TetR–FLAG–mCherry. **b**, Chromosomal distribution of the TetO integration sites. **c**, Relative ChIP–seq enrichment of indicated proteins at TetO platforms. Values are normalized to the Cherry condition. n.a., data not available for this condition. **d**, 4C overlay and CTCF ChIP–seq tracks of an example TetO locus. The plot is centered at TetO, as the 4C viewpoint. Common interactions are indicated in gray. **e**, Examples of the HMM TACL domains annotated based on the 4C-seq profiles of TACL-ON and Cherry cells. TetO platform marked in black and HMM domain highlighted in light blue. **f**, Average Hi-C interactions centered at all TetO integrations for TACL-ON and TACL-OFF conditions. Panel **a** created in BioRender. Han, R. (2025) https://BioRender.com/670827q.

**Extended Data Fig. 1:**
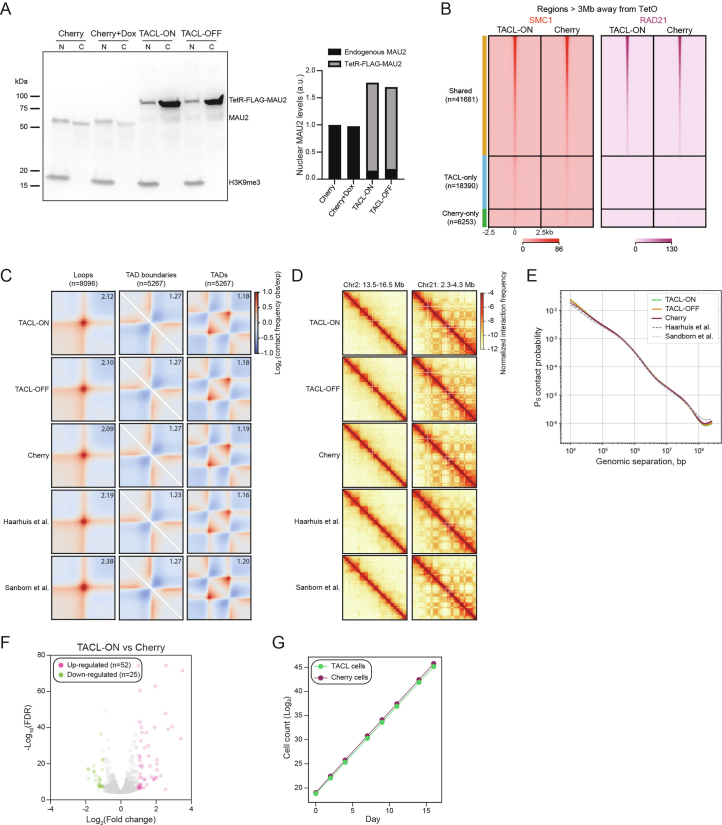
TetR-FLAG-MAU2 functionally replaces endogenous MAU2 and does not cause physiological changes in Hap1 cells. (**A**) Western blot analyzing the nuclear and cytoplasmic fractions of Cherry, Cherry+Dox, TACL-ON, and TACL-OFF cells (left panel). H3K9me3 signal is only present in the nuclear fraction (N), and is depleted from the cytoplasmic fraction (C). Right panel shows the quantification of MAU2 protein intensity levels the nuclear fraction, normalized to Cherry. (**B**) Tornado plots of ChIP-seq signals for SMC1 and RAD21 in TACL-ON and Cherry cells. Signal is shown at regions > 3 Mb away from the nearest TetO integration site. Peaks are divided into three groups; shared peaks between the two conditions, TACL-ON only, and Cherry-only. Note the similar levels between the two conditions across different genomic regions. (**C**) Average Hi-C loops (left), TAD boundaries (middle) and TADs (right) in TACL-ON, TACL-OFF, Cherry, and publicly available HAP1 cells^1,2^. Value in the upper-right corners indicates the average loop strength, average TAD boundary strength, and average TAD density over the background, respectively. (**D**) Hi-C interaction matrices at 100-kb resolution showing examples of chromatin structure in TACL-ON, TACL-OFF, Cherry, and publicly available HAP1 cells^1,2^ for Chr2: 135.0-165.0 Mb (left) and Chr21: 23.0-43.0 Mb (right). (**E**) Contact probability P(s) as a function of genomic separation for TACL-ON, TACL-OFF, Cherry, and publicly available HAP1^1,2^. Hi-C datasets, showing the global similarity between datasets. (**F**) Volcano plot showing differentially expressed genes measured by BrU-seq in TACL-ON vs. Cherry cells. Pink dots represent genes expressed at higher levels in TACL-ON cells while green dots represent genes with higher expression levels in Cherry cells. Y-axis represents −Log_10_ p-values while X-axis shows Log_2_ fold change values. (**G**) Cellular proliferation rates of TACL-ON and Cherry cells. Y-axis represents Log_2_ cell number of each cell line measured after indicated days on X-axis. Source data

We developed the TArgeted Cohesin Loader (TACL) system, a genetic platform for site-specific initiation and manipulation of individual loop extrusion trajectories in vivo. TACL uses the TetO/TetR system, introducing the TetR peptide fused to the cohesin loading factor MAU2 (SCC4) to conditionally recruit cohesin and initiate loop extrusion trajectories from Tet operator sequences integrated in the human genome. We used the PiggyBac transposon system^39^ to create a human HAP1 cell line with 27 TetO platforms (hereafter ‘TetO’) randomly inserted across 19 different chromosomes (Fig.1a,b). Each TetO platform contains 48 TetR binding sites. We chose HAP1 cells because they are haploid and therefore have no untargeted chromosome copies compromising the monitoring of TACL-induced effects. We wished to study the more general, non-anecdotal consequences of activated loop extrusion, and therefore typically performed aggregate analyses of 27 TetO sites instead of characterizing individually elected genomic insertions. Cells were transduced with lentivirus to stably express TetR fused to FLAG–MAU2 (‘TACL’) or to FLAG–mCherry (‘Cherry’). Western blotting showed that TetR–FLAG–MAU2 mainly localized to the cytoplasm, but also entered the nucleus, where it replaced most (~85%) endogenous MAU2 protein (Extended Data Fig. 1a). TetR–MAU2 competing with endogenous MAU2 for binding to NIPBL was not unexpected, as others previously found that NIPBL and MAU2 need each other for protein stability in the nucleus^22,40,41^. To assess whether TetR–MAU2 replacing endogenous MAU2 had any general impact, we studied cohesin distribution, chromosome topology and gene expression away from (>3 Mb) TetO sites. Chromatin immunoprecipitation followed by sequencing (ChIP–seq) for SMC1 and RAD21 showed that the distribution of cohesin along chromosomes was similar between TACL cells and control Cherry cells (Extended Data Fig. 1b). Hi-C also demonstrated that chromatin topology—that is, chromatin loops, TAD boundaries and TADs—was not altered in a meaningful way (Extended Data Fig. 1c–e). With nascent RNA sequencing, we observed only a few genes differentially expressed between TACL and Cherry cells (Extended Data Fig. 1f). Finally, the cellular proliferation rates of TACL and Cherry cells were comparable (Extended Data Fig. 1g). Therefore, we concluded that TetR–MAU2 was capable of functionally replacing endogenous MAU2 in HAP1 cells, without noticeably changing the overall cohesin biology of these cells.

### TACL recruits the cohesin complex to defined genomic locations

**Extended Data Fig. 2:**
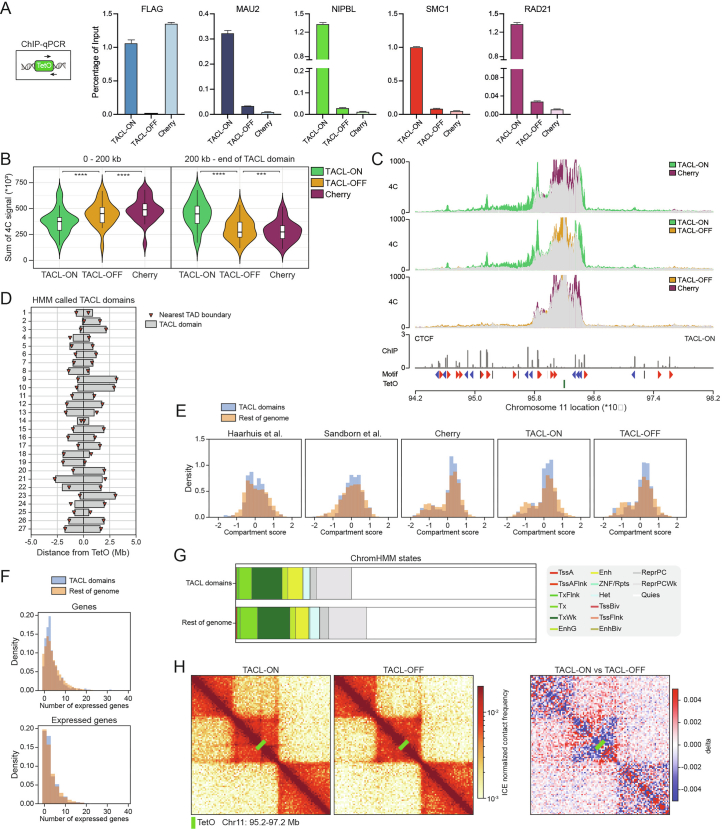
TACL efficiently recruits cohesin factors and induces topological changes on chromatin. (**A**) ChIP-qPCR, shown as percentage of input DNA, for the factors indicated in TACL-ON, -OFF, and Cherry cells. One set of primers was used to simultaneously detect all TetO platforms. (**B**) Violin plots summarizing the 4 C signals in TACL-ON, -OFF, and Cherry cells. TetO platforms were used as viewpoint for 4 C. 4 C signals are divided into two groups; 0-200 kb surrounding TetO platforms and 200-kb to the end of TACL domains. **** p value (paired t-test) < 0.0001; *** p value (paried t-test) <0.001. (**C**) Example 4 C overlay of a TetO integration on chr11 comparing TACL-ON, -OFF and Cherry conditions. (**D**) HMM called TACL domains (grey) centered at the TetO platform integration sites. Triangles indicate the closest annotated TAD border left and right of TetO. (**E**) Total, active gene densities, (**F**) compartment scores for Cherry, TACL-ON, TACL-OFF and publicly available HAP1 Hi-C data^1,2^ in 200 kb genomic bins overlapping with TACL domains and in the rest of the genome. Expressed genes were selected from the differential expression analysis of the BrU-seq data. (**G**) Chromatin states for HAP1 cells in TACL domains and in the rest of the genome. Previously annotated by ChromHMM states^3^ were used and lifted from the hg19 to the hg38 assembly using liftOver. (**H**) Hi-C interaction map of an example locus on chr11 in TACL-ON and TACL-OFF cells (two left plots). Right plot shows differential signal between TACL-ON vs. TACL-OFF for the same locus. The location of TetO is highlighted in green. Note the stripes emerging from the TetO platform.

Next, we investigated the consequences of TetR–MAU2 recruitment to the genomically integrated TetO sites. ChIP–qPCR with primers amplifying TetO sequences allowed the analysis of TetR–MAU2 recruitment to all TetO sites at once. We confirmed that the TetR–MAU2 and TetR–mCherry fusion proteins efficiently bound to TetO (Extended Data Fig. 2a). TetR binding to TetO was reversible: the TetR fusion proteins were released from TetO when cells were treated with doxycycline (Dox) for 1 h (Extended Data Fig. 2a). TetR–MAU2, not TetR–mCherry, selectively co-recruited NIPBL to TetO and attracted the core cohesin subunits SMC1 and RAD21, as seen previously in yeast^28^. Similarly, when we added Dox for 1 h, these factors were no longer recruited. This was confirmed in ChIP–seq experiments for all these factors (Fig. 1c and Extended Data Fig. 2a). We refer to the default, bound condition (no Dox) as ‘TACL-ON’, and to the induced, unbound condition (+Dox, 1 h) as ‘TACL-OFF’. The TetR–mCherry (Cherry) cells served as an alternative negative control condition.

When we pulled down chromatin associated with the cohesin subunits STAG1 and STAG2, we found that STAG2 was predominantly co-loaded onto TetO (Fig. 1c). This may show a preference of the system for cohesin^STAG2^ over cohesin^STAG1^, but could also be explained by the approximately fivefold higher RNA expression levels of STAG2 in HAP1 cells^42^. Collectively, the ChIP data showed that the targeting of MAU2 to TetO triggered the co-recruitment of NIPBL and core cohesin subunits. CTCF, PDS5A and WAPL, as well as cohesin-associating proteins, were not recruited by MAU2 to TetO (Fig. 1c).

### TACL enables local activation of cohesin loop extrusion

To test whether TACL enabled controlled activation of cohesin loop extrusion from TetO sites, we first performed 4C-seq to investigate changes in chromatin contacts made by the integrated TetO cassettes. Given that presumably each TetO predominantly contacts its own linear surrounding sequences, per condition, a single pair of 4C primers could be used to simultaneously assess the individual contact profiles of all 27 TetO locations. We observed that in the TACL-ON condition, all TetO sites engaged in more long-range contacts (>200 kb) at the expense of short-range contacts (Extended Data Fig. 2b). Clearly noticeable was that TACL induced many of the TetO platforms to form strong, specific interactions with surrounding CTCF sites, often over hundreds of kilobases (Fig. 1d and Extended Data Fig. 2c). The increased long-range contacts were absent in Cherry cells and completely dismantled in TACL-OFF cells (Fig. 1d,e and Extended Data Fig. 2c). The induced chromatin loops observed in TACL-ON cells strongly suggested that TACL enables controlled activation of chromatin loop formation from integrated TetO sites in living cells.

The gain in 4C signal in TACL-ON versus Cherry and TACL-OFF cells was used in a hidden Markov model (HMM) to define the domains with TACL-induced looping trajectories (TACL domains) (Fig. 1e; see Methods). These domains often extended asymmetrically from a TetO site and spanned an average genomic distance of 2.56 Mb (minimum, 1.08 Mb; maximum, 4.43 Mb) (Fig. 1e and Extended Data Fig. 2d). These distances fitted well with estimated cohesin loop extrusion ranges reported by others^43^. We assumed that they reflected the maximum lengths of TACL-induced cohesin loop extrusion trajectories, but they may also result from tandem loop extrusions mediated by multiple cohesin molecules. Together, these domains had representative (epi-)genomic features, with gene densities and active gene densities, compartment scores and ChromHMM states being similar to those seen elsewhere in the genome (Extended Data Fig. 2e–g).

### TACL induces TetO-anchored loop extrusion events

TACL-induced local 3D genome changes were further confirmed by Hi-C analysis. By averaging the chromatin contact data around all TetO sites, stripes, not chromatin jets, were seen emerging in a bi-directional manner from the TetO sites (Fig. 1f and Extended Data Fig. 2h). Chromatin jets (or fountains) describe a recently identified Hi-C signature observed at some locally dominant cohesin loading sites. They probably reflect bouquets of unanchored, bi-directionally extruding cohesin molecules that all initiated extrusion at the same site^43–47^. By contrast, stripes, normally observed at strong CTCF boundaries^15,48^, are believed to reflect differently sized chromatin loops as present across the cell population, all formed by unidirectional extruding cohesin molecules anchored at these sites. Our TACL system, particularly supporting unidirectional loop extrusion, may be because loops anchor at TetO, through the stable TetR–TetO interaction or through the loading of an impenetrable amount of cohesin complexes. Alternatively, TACL-loaded cohesin may first migrate and stall at TetO-flanking sites, to then reel in the TetO sequences (‘Discussion’).

### Cohesin transports NIPBL–MAU2 across looping trajectories

**Fig. 2:**
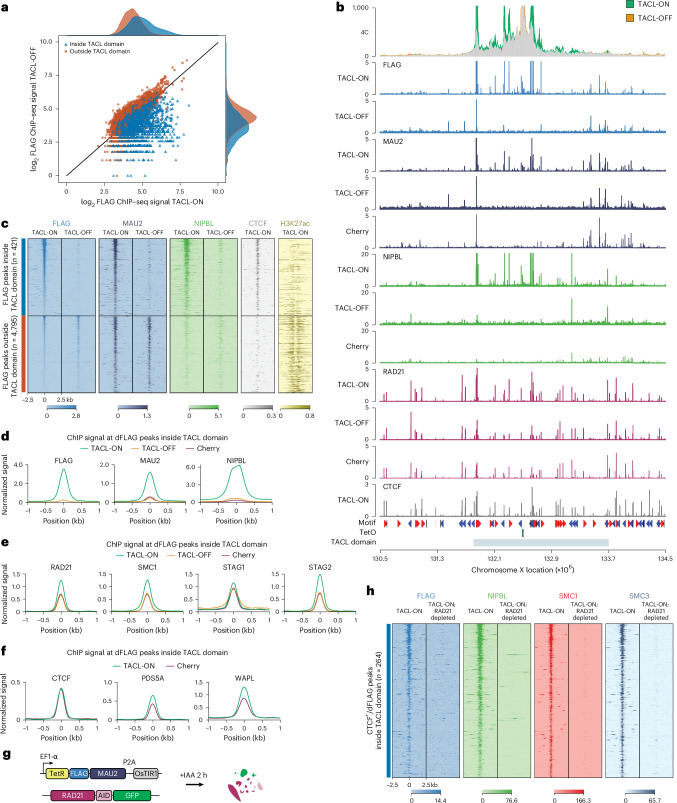
Local accumulation of the cohesin complex reveals important features of individual subunits. **a**, A log_2_(fold change) scatter plot of FLAG ChIP–seq signal in TACL-ON and TACL-OFF conditions. FLAG peaks inside or outside the TACL domain are depicted in blue or orange, respectively. Dox-responsive FLAG peaks are mostly located within the TACL domain. **b**, 4C overlay and ChIP–seq tracks of an example locus on chrX. TACL-OFF and Cherry tracks serve as controls. The plot is centered at TetO, being the 4C viewpoint. TACL domain indicates the HMM-determined loop extrusion domain. **c**, Tornado plots of ChIP–seq signals for cohesin loading factors, CTCF and H3K27ac. Signal is shown at FLAG peaks inside the HMM-defined TACL domain (blue), or outside the domain (orange). Peaks are plotted in ±2.5 kb windows. The color map indicates signal intensities. *n*, number of peaks. **d**–**f**, Aggregate signal plots showing normalized ChIP–seq signals for FLAG, MAU2 and NIPBL (**d**), for the core cohesin components RAD21, SMC1, STAG1 and STAG2 (**e**) and for CTCF, PDS5A and WAPL, at differential FLAG (dFLAG) peaks inside TACL domains (**f**). Signals are scaled to the average coverage at CTCF sites outside the TACL domains. **g**, Schematic illustration of the RAD21 degron system. **h**, Tornado plots of ChIP–seq signals for FLAG, NIPBL, SMC1 and SMC3 in TACL-ON and TACL-ON RAD21-depleted cells. Peaks shown are CTCF peaks inside the TACL domain overlapping with a dFLAG peak.

**Extended Data Fig. 3:**
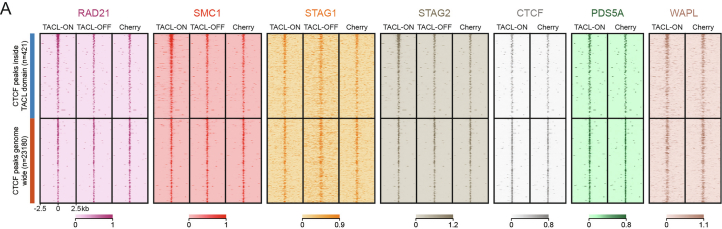
TACL induced the recruitment of cohesin-associated factors. (**A**) Tornado plots of ChIP-seq signals for cohesin associated factors as indicated, in TACL-ON, -OFF, and Cherry cells. Signal is shown at CTCF peaks inside the HMM defined TACL domain (blue), or outside the domain (orange). Peaks are plotted in ± 2.5 kb windows. The color map indicates signal intensities. ‘n’ stands for the number of peaks.

To understand whether TACL reshaped the distribution of cohesin and its interaction partners across TACL domains, we performed ChIP–seq experiments, beginning with FLAG-tagged TetR–MAU2. Although TetR–MAU2 was broadly distributed across the genome, the subset of binding sites that responded to Dox and disappeared in TACL-OFF cells were specifically localized within the TACL domains (Fig. 2a,b). Outside these domains, MAU2 was primarily associated with active enhancers (Fig. 2c). Within TACL domains, however, MAU2 and NIPBL selectively accumulated at CTCF sites in a TACL-dependent manner (Fig. 2b–d). These CTCF sites were pre-existing: in control Cherry cells, they functioned to stall naturally loaded cohesin complexes and co-recruited PDS5A and WAPL (Extended Data Fig. 3a). In TACL-ON cells, the binding of cohesin, PDS5A and WAPL to these CTCF sites increased (Fig. 2e,f). Thus, TACL supported the transport of cohesin and MAU2–NIPBL from TetO to flanking CTCF sites. Probably as a consequence, these CTCF sites now also bound more PDS5A and WAPL.

**Extended Data Fig. 4:**
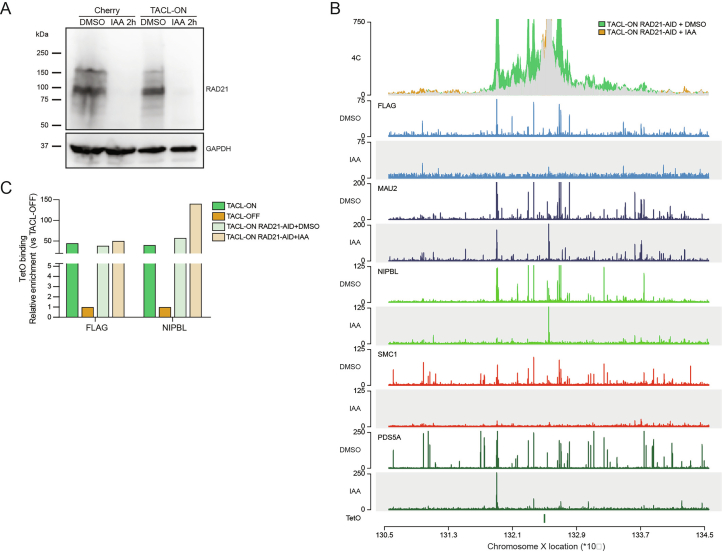
TACL-induced loop extrusion is RAD21 dependent. (**A**) Western blot analysis of RAD21 in Cherry and TACL-ON RAD21-AID cells. DMSO serves as a control. RAD21 is degraded by 2 h of IAA treatment. GAPDH is used as loading control. (**B**) An example locus on chrX presenting the 4 C overlay and ChIP-seq tracks of FLAG, MAU2, NIPBL, SMC1, and PDS5A in TACL-ON RAD21-depleted cells or non-depleted cells (DMSO). The plot is centered at the 4 C viewpoint indicated as TetO in the lower part of the panel. (**C**) Relative enrichment of FLAG and NIPBL at the TetO platforms, calculated form ChIP-seq data. Values are normalized to TACL-OFF condition. Note the unchanged/increased binding of the factors in RAD21-depleted cells. Source data

Given that NIPBL and MAU2 were not known to associate with CTCF sites, their TACL-induced deposition at CTCF sites was surprising (Fig. 2c,d). To investigate whether this was a consequence of their high concentration at TetO sites and subsequent nucleoplasmic diffusion or of their co-migration with cohesin extruding from TetO, we knocked in an auxin-inducible degron (AID2)^49^ to acutely deplete endogenous RAD21 in the TACL and Cherry cells (Fig. 2g). Treating the TACL cells for 2 h with 5-Ph-IAA (IAA) degraded RAD21 to undetectable levels (Extended Data Fig. 4a) and dismantled the TACL-induced topological contacts, as determined by 4C-seq (Extended Data Fig. 4b). TetR–MAU2 and NIPBL were still efficiently recruited to TetO (Extended Data Fig. 4c) but were no longer detected at the flanking CTCF sites, where SMC1 and SMC3 were also removed (Fig. 2h). This strongly suggests that MAU2 and NIPBL accumulated at flanking CTCF sites through co-migration with, and subsequent pausing of, TACL-induced loop-extruding cohesin complexes.

**Fig. 3:**
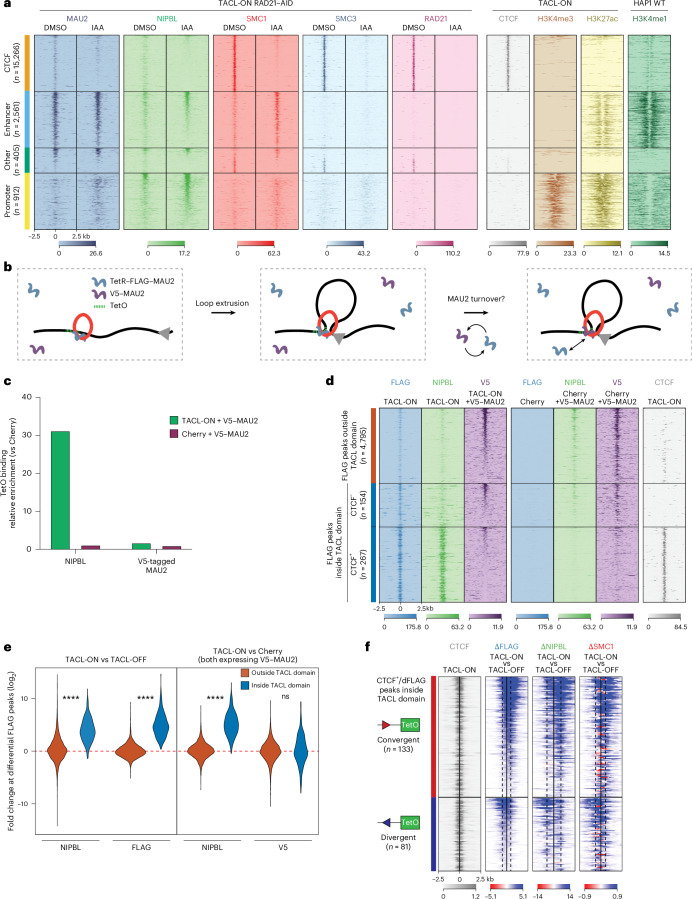
MAU2 forms a stable extruding complex with cohesin. **a**, Tornado plots of ChIP–seq signals for MAU2, NIPBL, SMC1, SMC3 and RAD21 in TACL-ON RAD21-AID cells. For reference, CTCF, H3K4me3, H3K27ac and H3K4me1 signals are displayed. SMC1 and NIPBL positive peaks located outside TACL domains and >3 Mb from the TetO integration site are categorized into four groups as indicated on the left: CTCF, enhancer (H3K4me3^−^/H3K27ac^+^ and/or H3K4me1^+^), other (no active histone modification marks) and promoter (H3K4me3^+^). *n*, number of peaks; WT, wild type. **b**, Schematic representation of the TetR–FLAG–MAU2 and V5–MAU2 co-expression experiment. **c**, Relative ChIP–seq enrichment of NIPBL and V5–MAU2 at TetO platforms. Values are normalized to the Cherry condition. **d**, Tornado plots of ChIP–seq signals for FLAG, NIPBL and V5 in TACL-ON and Cherry cells co-expressing V5–MAU2. FLAG peaks in TACL-ON cells are divided into three categories (shown on the left): outside the TACL domain, inside the TACL domain positive for CTCF and inside the TACL domain negative for CTCF. *n*, number of FLAG peaks. **e**, Violin plots showing log_2_(fold change) at FLAG peaks inside (blue) and outside (orange) TACL domains overlapping with CTCF sites in TACL-ON cells. The left panel compares NIPBL and FLAG signals between TACL-ON and TACL-OFF; the right panel compares NIPBL and V5 signals between TACL-ON and Cherry cells co-expressing V5–MAU2. Statistical significance determined using the Mann–Whitney *U*-test: ****P < 0.0001. ns, not significant. For TACL-ON versus TACL-OFF: NIPBL *P* = 1.46 × 10^−102^, FLAG, *P* = 3.25 × 10^−^^156^. For TACL-ON versus Cherry (expressing V5–MAU2), NIPBL *P* = 6.01 × 10^−^^152^, V5 *P* = 2.52 × 10^−^^1^. **f**, ChIP–seq tornado plots of CTCF, and differential signal for FLAG, NIPBL and SMC1 between TACL-ON and TACL-OFF conditions. Peaks shown are CTCF peaks inside the TACL domain overlapping with a dFLAG peak. Upper half (red bar) shows CTCF sites with a convergent orientation towards TetO; lower half (blue bar) shows CTCF sites with a divergent orientation towards the TetO platform. *n*, number of CTCF sites. The color map indicates a blue signal as enrichment in TACL-ON over TACL-OFF, and vice versa for red.

**Extended Data Fig. 5:**
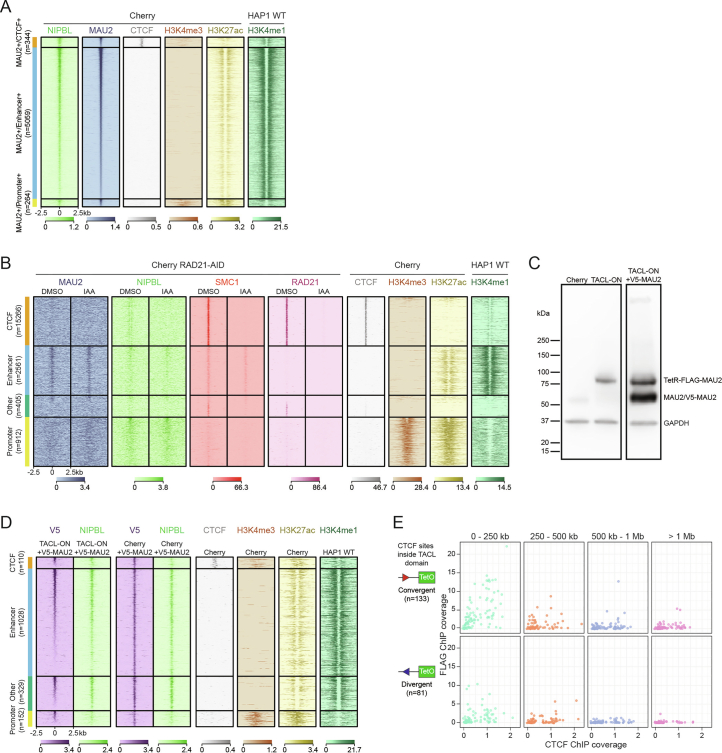
MAU2 is stably associated with cohesin from start to the end. (**A**) Tornado plots of ChIP-seq signals for NIPBL, MAU2, CTCF, H3K4me3, and H3K27ac in Cherry cells. Publicly available H3K4me1 ChIP-seq in WT HAP1 cells (ENCODE: ENCSR450JTP) is included for reference. Genome-wide MAU2 ChIP-seq peaks in Cherry cells are divided into three groups based their co-localization with CTCF, enhancer (H3K4me3- and H3K4me1 or H3K27ac+), or promoter (H3K4me3+) histone marks. Peaks are plotted in ± 2.5 kb windows. The color map indicates signal intensities. ‘n’ stands for the number of peaks. (**B**) Tornado plots of ChIP-seq signals for MAU2, NIPBL, SMC1, and RAD21 in Cherry RAD21-AID cells. CTCF, H3K4me3, H3K27ac, and H3K4me1 signals from Cherry cells are also displayed to indicate the four different groups as shown on the left; CTCF, Enhancer (H3K4me3- and H3K27ac+ or H3K4me1+), Other (no active histone modification marks), and Promoter (H3K4me3+). The color map indicates signal intensities. ‘n’ stands for the number of peaks. (**C**) Western blot analysis of MAU2 in Cherry, TACL-ON, and TACL-ON + V5-MAU2 cells. Different versions of MAU2 are visualized on the same blot with MAU2 antibody. GAPDH serves as loading control. (**D**) Tornado plots of ChIP-seq signals for V5 and NIPBL in TACL-ON and Cherry cells ± V5-MAU2 co-expression. For reference, CTCF, H3K4me3, H3K27ac, and H3K4me1 signals are displayed. V5 peaks are classified into four groups as mentioned above. (**E**) Correlation of FLAG and CTCF ChIP-seq coverage signals. ChIP-seq signals are grouped based on their distances and CTCF orientation relative to TetO. Source data

To investigate whether the cohesin-mediated transport of NIPBL–MAU2 along chromosomes to CTCF sites was a peculiarity of TACL or also happened naturally, we re-analyzed the ChIP–seq data. As also reported by others^43,45^, when we called peaks in our NIPBL and MAU2 ChIP–seq datasets from control Cherry cells, they typically overlapped with active promoters and enhancers, not at CTCF sites (Extended Data Fig. 5a). However, when we selected and combined all SMC1 and NIPBL binding sites and stratified them according to co-localization with CTCF sites, enhancers or promoters, we found that NIPBL and MAU2 were also enriched at many CTCF sites across the genome. This was emphasized in TACL cells that overexpressed the MAU2 fusion protein (Fig. 3a) but was also appreciable in control Cherry cells (Extended Data Fig. 5b). When we depleted RAD21 from TACL and control Cherry cells, NIPBL and MAU2, but also SMC1, remained associated with enhancers and promoters but disappeared from CTCF sites (Fig. 3a and Extended Data Fig. 5b). This demonstrated that, in wild-type cells as well, NIPBL and MAU2 were brought by cohesin to CTCF sites. Our observation that without RAD21, NIPBL–MAU2 and SMC1 remained associated with enhancers, while others reported that NIPBL–MAU2 disappeared from nearly all its binding sites, may be explained by our measurements being taken immediately (2 h) after RAD21 protein depletion, instead of 48 h after RNAi depletion^31^.

Previous in vitro experiments suggested that the extruding cohesin complex transiently associated with NIPBL, and presumably MAU2, and that a dynamic exchange of NIPBL (and MAU2) was needed for cohesin to act as an active loop-extruding holo-enzyme^19,20^. To study this exchange in vivo, we co-expressed V5-tagged MAU2 with FLAG-tagged TetR–MAU2 in TACL cells (Fig. 3b). Western blotting with a MAU2 antibody suggested that the expression of V5-tagged MAU2 was higher than that of the FLAG-tagged TetR–MAU2 protein (Extended Data Fig. 5c). ChIP–seq demonstrated that outside of TACL domains, V5–MAU2 occupied the same sites as MAU2 (and TetR–MAU2), being mostly active at promoters and enhancers, but also weakly active at CTCF sites (Extended Data Fig. 5d). However, V5–MAU2 had no affinity for, and was not recruited to, TetO (Fig. 3c) and it also remained absent from the TetO-flanking CTCF sites (Fig. 3d,e). We therefore found no evidence for exchange of MAU2 during loop extrusion in TACL cells. This discrepancy with published in vitro data^19,20^ may be because NIPBL and MAU2 differ in their binding kinetics or stability on cohesin, or simply because the local TetR–MAU2 protein concentrations around TetO were too high for MAU2–V5 to effectively compete in the exchange. Alternatively, this finding may be because we examined anchored cohesin loop extrusion in vivo, on chromatinized DNA, whereas the in vitro assays studied unanchored cohesin loop extrusion on naked DNA.

### Cohesin traffic jams at blocking CTCF sites

Given that CTCF binding orientation on DNA is important for halting cohesin loop extrusion^23–25^, we examined whether the blocking sites were facing towards (convergent) or away (divergent) from TetO. As expected, it was predominantly the CTCF sites facing TetO that most effectively blocked TACL-induced loop extrusion (Extended Data Fig. 5e). Interestingly, however, divergent CTCF sites (looking away from TetO) also accumulated cohesin as a consequence of TACL (Fig. 3f). Closer inspection revealed that TACL-induced cohesin accumulation at convergently oriented CTCF sites led to queuing of multiple extruding cohesin holoenzymes in front of, but also behind, the CTCF sites. Similar queues were also present in front of and behind divergently bound CTCF molecules (Fig. 3f). This pattern suggests that extruding cohesin complexes were halted upon encountering another paused cohesin stalled at a CTCF-bound site, forming in vivo cohesin traffic jams. Such cohesin traffic jams became visible by ChIP–seq, probably because TACL triggers high local loop extrusion activity to saturate extrusion trajectories and amplify stalling events at individual sites. Although in vitro studies have shown that purified condensin complexes can pass each other during loop extrusion on naked DNA^50^, in vivo cohesin ‘traffic jams’ have been hypothesized based on the observation of loop collision events^51–53^. We speculate that the traffic jams observed with TACL may also occur naturally, but at stochastic intervals and genomic locations, making them difficult to be detected at individual sites in wild-type cells.

### Modifying the lengths of loop extrusion trajectories

**Fig. 4:**
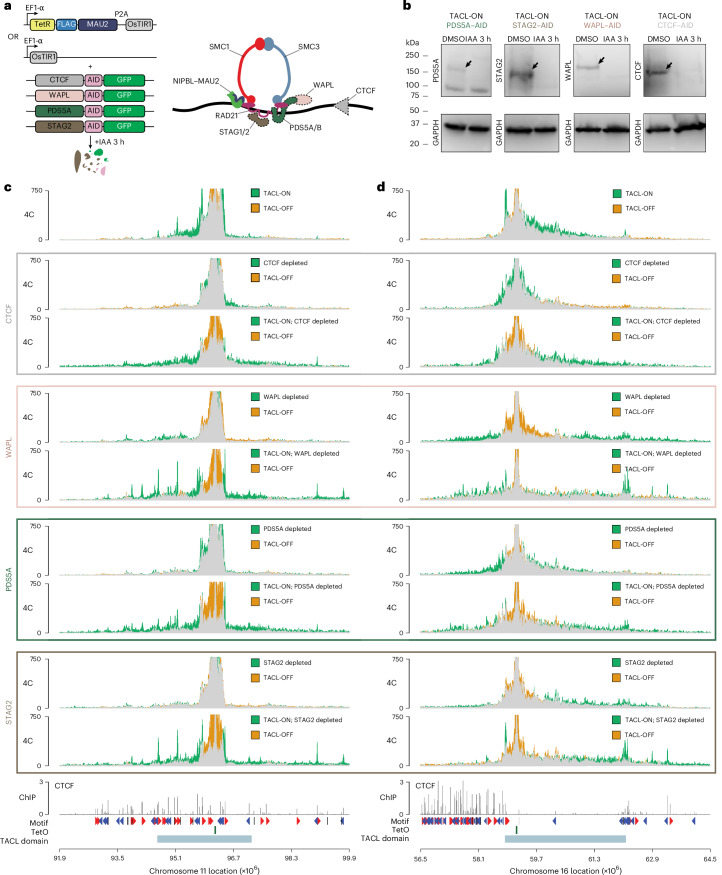
Depletion of cohesin factors causes loop extension. **a**, Schematic overview of inducible degron systems for CTCF, WAPL, PDS5A and STAG2 (left), and their localization on the cohesin complex (right). For each degron, a cell line was generated with TetR–FLAG–MAU2 + OsTir1 expression and a cell line with only OsTir1 expression. **b**, Western blot images of degrons before and after depletion. Proteins were depleted by 3 h of IAA and detected using the corresponding antibodies. GAPDH was used as a loading control. Western blot was performed in duplicate, with the same results. **c**,**d**, 4C chromatin contact tracks of two example TetO sites, one on chromosome 11 (**c**) and the other on chromosome 16 (**d**). For reference, the plots on top again show a comparison of the chromatin interactions formed by TetO in TACL-ON and TACL-OFF cells. The plots below show for CTCF, WAPL, PDS5A and STAG2 how their depletion affects the TetO chromatin interactions, when depletion is performed in cells without TACL and in TACL-ON cells. By comparing the latter chromatin interaction profiles (TACL-ON; factor depleted) with the reference TACL-ON chromatin interaction profile shown on top, one can appreciate the impact of factor depletion on TACL-induced chromatin interactions. Source data

**Extended Data Fig. 6:**
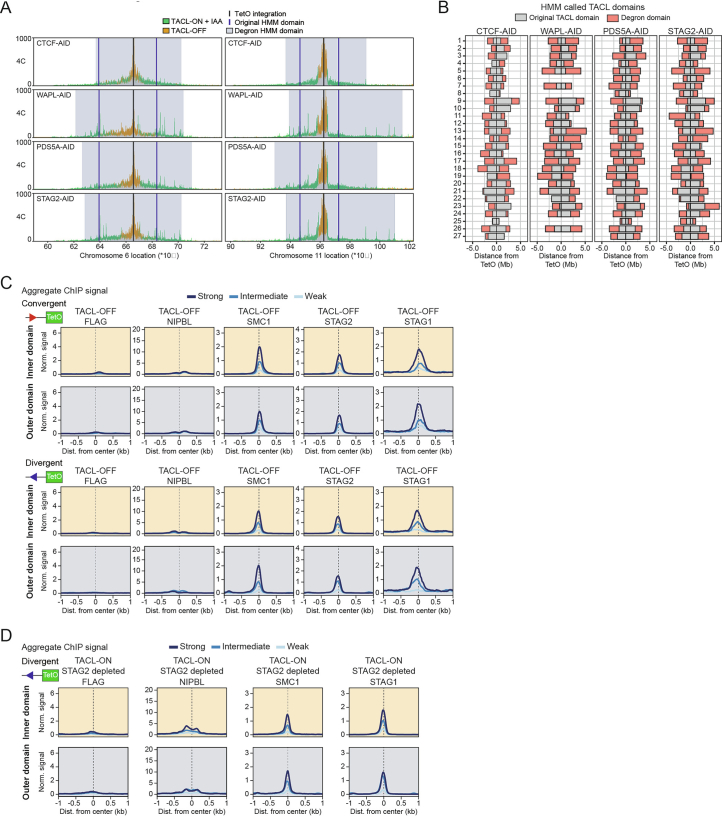
Depletion of cohesin factors cause extended loop extrusion. (**A**) 4 C overlays at two example loci in different degron lines. Comparisons are made between TACL-OFF and TACL-ON + depletion (IAA). 4 C plots are centered around TetO. Original TACL domain are depicted by dark blue lines and the extended domain after degron depletion is depicted by the grey box. Note the extension of domains after depletion. (**B**) HMM called TACL domains (grey) and extended degron domains (orange) for each degron cell line after depletion. Plots are centered at the TetO platform integration sites. (**C**) Average signal plots of FLAG, NIPBL, SMC1, STAG2, and STAG1 ChIP-seq signals at CTCF sites within the inner or outer TACL domains. CTCF sites are categorized based on orientation to TetO and CTCF strength. Peaks are plotted as ± 1 kb windows centered on highest ChIP-seq signals. ChIP signals are normalized to the average signal at CTCF sites genome wide. Number of sites in the inner domain are; convergent-strong n = 77; convergent-intermediate n = 65; convergent-weak n = 59; divergent-strong n = 78; divergent-intermediate n = 57; divergent-weak n = 53. Number of sites in the outer domain are; convergent-strong n = 48; convergent-intermediate n = 42; convergent-weak n = 35; divergent-strong n = 32; divergent-intermediate n = 38; divergent-weak n = 41. (**D**) Average signal plots of FLAG, NIPBL, SMC1, and STAG1 ChIP-seq signals for TACL-ON STAG2 depleted cells for TetO convergent CTCF sites. Signal is shown at CTCF sites within the inner or outer TACL domains. CTCF sites are categorized based on CTCF strength. Number of sites as indicated above. Peaks are plotted as ± 1 kb windows centered on highest ChIP-seq signals. ChIP signals are normalized to the average signal at CTCF sites genome wide.

PDS5A is thought to restrict chromatin loop sizes by competing with and displacing NIPBL from cohesin^3,29,30^. Cohesin^STAG2^ associates less stably with chromatin than cohesin^STAG1^ (refs. ^11–13^). WAPL releases cohesin from chromatin^22^. Previous Hi-C studies have shown that depletion of WAPL^22^, PDS5A^54^ and STAG2 (ref. ^11^) resulted in increased CTCF–CTCF contacts over larger distances, suggesting extended loop extrusion trajectories. To explore this in our TACL system, we generated inducible degron cell lines for CTCF, WAPL, PDS5A and STAG2 in TetO-containing HAP1 cells with and without TetR–MAU2 expression (Fig. 4a,b). Without TetR–MAU2 expression, the depletion of each factor had a mild impact on the chromatin contacts of the unbound TetO platforms: loss of WAPL, STAG2 and PDS5A engaged the platforms in slightly more but non-specific contacts over increased distances, while loss of CTCF, if anything, had an opposite effect (Fig. 4c,d). Topological changes were much more pronounced when we depleted these proteins from TACL-ON cells (Fig. 4c,d). As expected, without CTCF, the TetO sites no longer formed specific loops with flanking CTCF sites (Fig. 4c,d). Without WAPL, the TetO sites also formed less frequent contacts with proximal CTCF sites but now engaged in new, prominent interactions with more distal CTCF anchors, in some cases over 4–5 Mb away (Fig. 4c,d). STAG2 and PDS5A depletion similarly affected these TetO chromatin contacts, although PDS5A depletion induced a milder effect than WAPL or STAG2 (Fig. 4c,d). Consequently, all depletions resulted in much larger TACL domains, as confirmed by HMM-based analysis of differential 4C contact maps (Extended Data Fig. 6a,b). Although the average TACL domain size was 2.55 Mb, it increased to 6.39 Mb (maximum, 8.23 Mb), 5.91 Mb (maximum, 7.98 Mb) and 5.59 Mb (maximum, 8.39 Mb) in WAPL-depleted, STAG2-depleted and PDS5A-depleted cells, respectively.

### CTCF site selection for chromatin looping

**Fig. 5:**
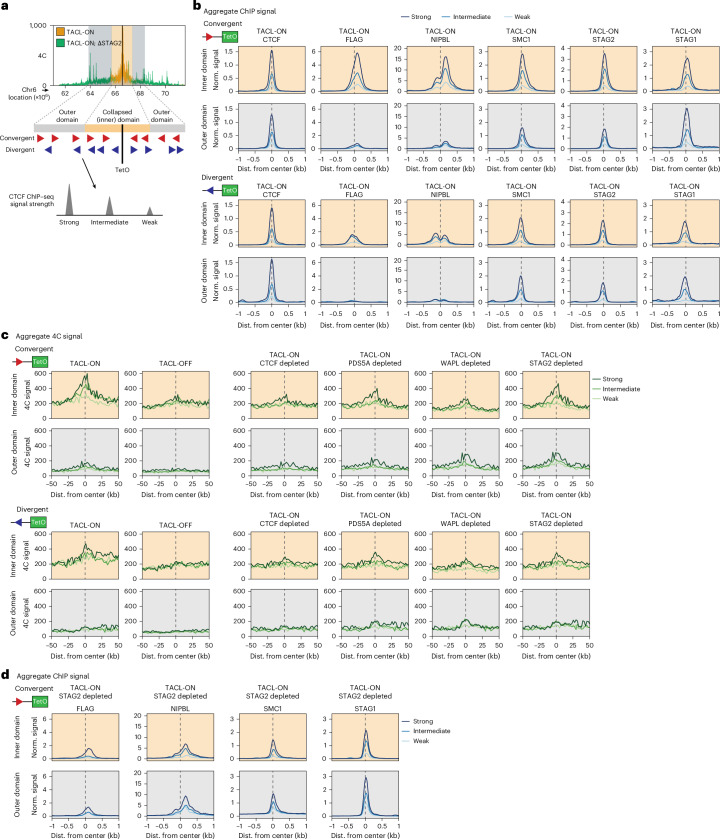
Complex CTCF contact networks are altered upon depletion of cohesin factors. **a**, Representation of the collapsed ‘inner’ domain in STAG2-depleted cells (highlighted in orange) as determined from 4C and HMM modeling. The outer TACL domain runs until the original TACL domain boundaries. CTCF binding sites within these domains are categorized by orientation (convergent or divergent relative to TetO) and ChIP–seq signal strength (strong, intermediate or weak). **b**, Average signal plots of CTCF, FLAG, NIPBL, SMC1, STAG2 and STAG1 ChIP–seq signals at CTCF sites within the inner or outer TACL domains. CTCF sites are categorized by orientation to TetO and CTCF strength. Peaks are plotted as ±1 kb windows centered on the highest ChIP–seq signals. ChIP signals are normalized to the average signal at CTCF sites genome-wide. Number of sites in the inner domain: convergent-strong, *n* = 77; convergent-intermediate, *n* = 65; convergent-weak, *n* = 59; divergent-strong, *n* = 78; divergent-intermediate, *n* = 57; divergent-weak, *n* = 53. Number of sites in the outer domain: convergent-strong, *n* = 48; convergent-intermediate, *n* = 42; convergent-weak, *n* = 35; divergent-strong, *n* = 32; divergent-intermediate, *n* = 38; divergent-weak, *n* = 41. **c**, Aggregate 4C signal plots of TACL-ON, TACL-OFF and TACL-ON degron depleted cells. Signal is plotted at CTCF sites within the inner and outer TACL domains. Again, CTCF sites are categorized by orientation to TetO and strength. Signals are centered (gray dashed line) around a ±50 kb window. Number of sites in the inner domain: convergent-strong, *n* = 57; convergent-intermediate, *n* = 58; convergent-weak, *n* = 49; divergent-strong, *n* = 63; divergent-intermediate, *n* = 44; divergent-weak, *n* = 44. Number of sites in the outer domain: convergent-strong, *n* = 48; convergent-intermediate, *n* = 45; convergent-weak, *n* = 35; divergent-strong, *n* = 32; divergent-intermediate, *n* = 39; divergent-weak, *n* = 41. **d**, Average signal plots of FLAG, NIPBL, SMC1 and STAG1 ChIP–seq signals in TACL-ON STAG2-depleted cells. Signal is plotted at TetO-convergent CTCF sites within the inner or outer TACL domains. Sites are categorized based on CTCF strength. Peaks are plotted as ±1 kb windows centered on the highest ChIP–seq signals. Number of sites as indicated in **b**.

Depletion of each factor also resulted in a dramatic collapse of local intradomain contacts across all TACL domains. We used HMM, based on the 4C-seq contact differences after STAG2 depletion, to define the collapsed ‘inner TACL domains’ and the intact ‘outer TACL domains’ (Fig. 5a). Within these domains, we distinguished three categories of CTCF sites based on their ChIP–seq signal strength: strong, intermediate and weak CTCF binding sites. We used the orientation of their CTCF binding motif to further separate them into TetO-convergent and TetO-divergent CTCF sites (Fig. 5a). Figure 5b shows that TACL mainly deposited cohesin at the convergently oriented, but also at the divergently oriented CTCF sites. Furthermore, STAG1 behaved exceptionally as it bound more to distal (in outer domains) than proximal (in inner domains) CTCF sites (Fig. 5b). This is consistent with cohesin^STAG1^ forming more extended loop extrusion trajectories than cohesin^STAG2^.

To investigate how these different categories of CTCF sites contributed to TACL-induced loop formation, we designed an aggregate 4C analysis based on the TetO-centered 4C-seq contact profiles. We extracted 4C signals within ±50 kb of each previously defined CTCF site and grouped them per category of CTCF binding sites. As expected, in TACL-OFF cells, none of the categories showed preferential contacts with TetO. In TACL-ON cells, however, the TetO-convergent CTCF sites in the inner and outer TACL domains all engaged in looping contacts with TetO. These interactions were even detectable at the weak CTCF binding sites (Fig. 5c). Indeed, the local divergent CTCF sites also engaged in specific contacts with TetO (Fig. 5c). As these CTCF sites also accumulated NIPBL and FLAG-tagged MAU2, with NIPBL piling up on both sides of the TetO-divergent CTCF sites (Fig. 5b and Extended Data Fig. 6c), this seems to be further evidence of TACL causing cohesin traffic jams and loop collisions at surrounding CTCF sites. Therefore, by analyzing a large number of CTCF sites for their collective ability to form loops with defined, highly active chromatin loop extrusion anchors, we found that most CTCF sites can engage in temporarily stabilized loops with other extruding anchors. This revealed that unexpectedly complex CTCF contact networks can exist inside contact domains.

### TACL combined with the depletion of loop extrusion modifiers

We then asked how the loss of CTCF, WAPL, PDS5A and STAG2 affected these CTCF contact networks. Unsurprisingly, CTCF depletion abolished looping of all CTCF sites to TetO (Fig. 5c). WAPL depletion caused a local dismantling of TetO contacts but simultaneously stimulated TetO contacts with more distal CTCF sites. This was even seen for the distal divergent sites (Fig. 5c). Therefore, as previously observed^22^, the loss of WAPL increased loop formation with non-convergent CTCF sites. Our data suggested that this may be the consequence of cohesin queuing and loop collisions at these CTCF sites. Depletion of PDS5A disrupted all TetO contacts with local CTCF sites, but contact with the more distal, strongest CTCF sites seemed to be stimulated (Fig. 5c).

Finally, in STAG2-depleted cells, local TetO–CTCF contacts were partially destabilized, indicating that cohesin^STAG1^ cannot fully substitute for cohesin^STAG2^ in maintaining these interactions. Yet without cohesin^STAG2^, cohesin^STAG1^ more frequently engaged TetO in interactions with distal CTCF sites (Fig. 5c), where it also deposited more FLAG-tagged MAU2, NIPBL and SMC1 (Fig. 5d and Extended Data Fig. 6d). These results suggest that cohesin^STAG1^ exhibits higher loop extrusion processivity, which is hindered by other cohesin complexes under STAG2-proficient conditions.

### High cohesin loop extrusion activity hinders transcription

**Fig. 6:**
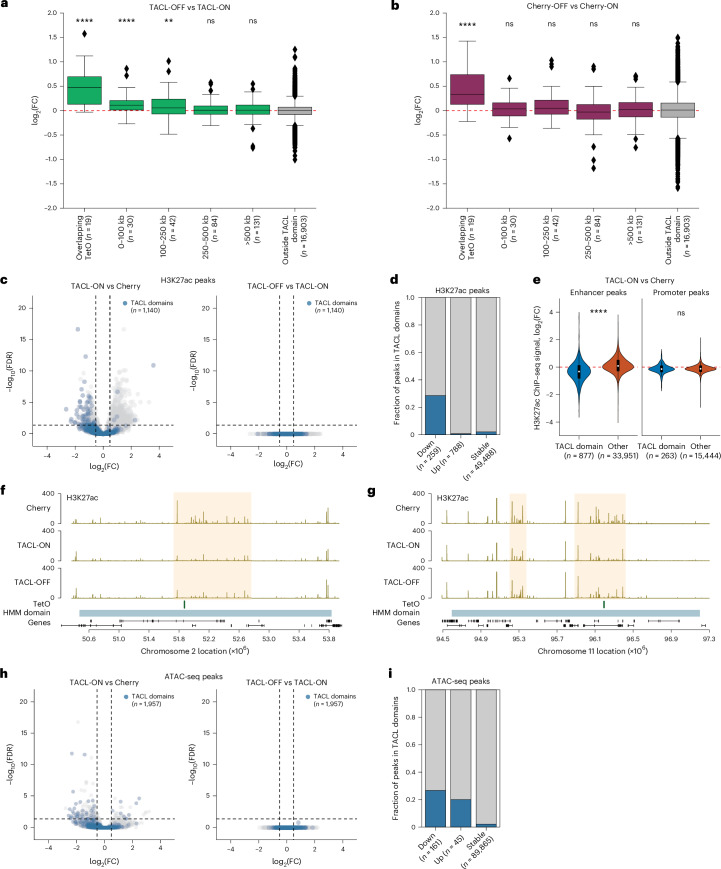
TACL-induced cohesin loop extrusion alters transcription and histone modifications. **a**,**b**, Relative gene expression changes in TACL-OFF versus TACL-ON (**a**) or Cherry-OFF versus Cherry-ON cells (**b**). OFF conditions were treated with Dox for 2 h. *Y* axes are plotted as log_2_(fold change (FC)) between conditions. Genes within the TACL domains are divided into different groups based on their distances to TetO. The number of genes for each group is indicated in parentheses. Each group of genes is compared to the control group ‘outside TACL domain’. The *P* values were corrected for multiple testing using the Benjamini–Hochberg (false discovery rate, FDR) procedure, yielding *q* values. Significance is indicated as ****q < 0.0001 and **q < 0.01 for two-sided Mann–Whitney *U*-tests. For TACL-OFF versus TACL-ON: *q* = 5.1 × 10^−8^ (overlapping TetO), *q* = 7.3 × 10^−^^5^ (0–100 kb), *q* = 0.01 (100–250 kb), *q* = 0.43 (250–500 kb), *q* = 0.07 (>500 kb). For Cherry-OFF versus Cherry-ON: *q* = 0.0007 (overlapping TetO), *q* = 0.75 (0–100 kb), *q* = 0.17 (100–250 kb), *q* = 0.26 (250–500 kb) and *q* = 0.82 (>500 kb). Horizontal lines in the boxplots correspond to the median, the box extends between the first (Q1) and the third quartile (Q3) and the error bars represent the interquartile range. **c**, Volcano plots showing differential H3K27ac peaks in TACL-ON versus Cherry cells or TACL-OFF versus TACL-ON cells. A total of 1,140 peaks are identified within the TACL domain (colored blue). **d**, Fraction of the differential H3K27ac peaks that lie within TACL domains (blue) out of all differential H3K27ac peaks (gray). **e**, Violin plots showing log_2_(FC) of H3K27ac peaks at enhancer and promoter sites in TACL-ON and Cherry cells. H3K27ac peaks are categorized into either inside (blue) or outside (orange) TACL domain. ****P < 0.0001 for two-sided Mann–Whitney *U*-test. For H3K27ac peaks at enhancers, *P* < 2.2 × 10^−16^; for H3K27ac peaks at promoters, *P* = 0.51. Violin plots represent the distribution of the signal in all H3K27ac peaks. Horizontal lines of the boxplots inside the violin plot correspond to the median, the box extends between Q1 and Q3 and the error bars represent the interquartile range. **f**,**g** H3K27ac ChIP–seq tracks in Cherry, TACL-ON and TACL-OFF cells of two example TetO sites on chromosome 2 (**f**) and chromosome 11 (**g**). Decreased H3K27ac levels in TACL-ON cells are highlighted by the yellow box. **h**, Volcano plots showing differential ATAC peaks in TACL-ON versus Cherry cells or TACL-OFF versus TACL-ON cells. A total of 1,957 peaks are identified within the TACL domain (colored blue). **i**, Fraction of the differential ATAC-seq peaks that lie within TACL domains (blue) out of all differential ATAC-seq peaks (gray).

We then investigated whether TACL-induced loop extrusion had an impact on the transcription of genes surrounding TetO. We measured and compared nascent transcription in TACL-ON and TACL-OFF cells, as well as in Cherry cells treated with and without Dox (2 h). A total of 19 active genes had a TetO platform integrated in their gene body (often seen with PiggyBac insertions^55^): when TetR–MAU2 or TetR–mCherry was released from their gene body (with Dox), expression of these genes was upregulated (Fig. 6a). The remaining genes located within the TACL domains (that is, the genes not having TetO integrated in their gene body) were categorized based on their distance to TetO. None of these categories of genes responded to TetR–mCherry release from TetO. However, the genes that were located within 250 Kb from TetO showed, on average, an increase in transcription in TACL cells when we stopped targeted loop extrusion by Dox addition (Fig. 6a,b). This finding suggests that transcription of genes by the RNA polymerase II machinery is hindered by increasing numbers of bypassing (or halting) extruding cohesin complexes.

### TACL alters local chromatin accessibility and H3K27ac levels

**Extended Data Fig. 7:**
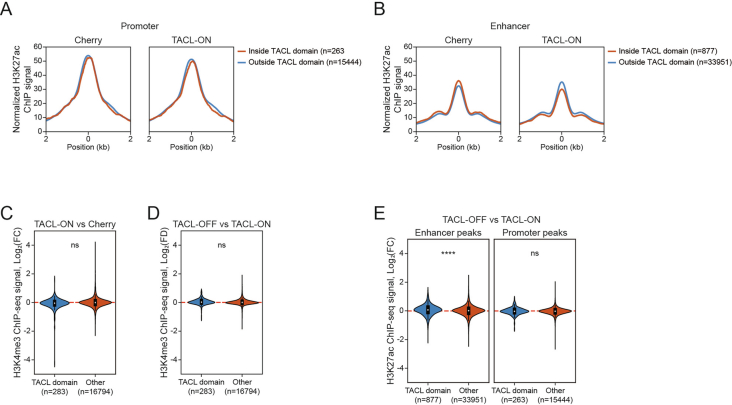
TACL-induced loop extrusion impact active histone modifications. (**A**, **B**) Average signal plots for H3K27ac ChIP-seq signals at enhancers (**C**) and promoters (**D**), in TACL-ON and Cherry cells. H3K27ac peaks are divided into either inside TACL domain (blue) or outside TACL domain (orange). The number of peaks is depicted between brackets of each category. (**C**, **D**) Violin plots showing Log_2_ fold change (FC) of H3K4me3 peaks in TACL-ON vs. Cherry (**A**), and TACL-OFF vs. TACL-ON (**B**) cells. H3K4me3 peaks are categorized into either inside (blue) or outside (orange) TACL domain. ‘ns’ indicates not significant. (**E**) Violin plots showing Log_2_ FC of H3K27ac peaks at enhancer and promoter sites in TACL-ON and TACL-OFF cells. H3K27ac peaks are categorized into either inside (blue) or outside (orange) TACL domain. **** p value < 0.0001; ‘ns’ stands for not significant.

Finally, we used TACL to investigate whether locally induced chromatin loop extrusion alters local chromatin accessibility and the epigenetic landscape. For this analysis, we performed assay for transposase-accessible chromatin using sequencing (ATAC-seq) and ChIP–seq for the active histone marks H3K27ac and H3K4me3, in TACL-ON and Cherry control cells. When analyzing H3K27ac signals, we identified 259 lost and 788 gained H3K27ac peaks (out of >50,000) in TACL-ON compared to Cherry cells. The sites with increased H3K27ac were not enriched inside TACL domains, but sites with lower levels of H3K27ac were highly enriched in the TACL domains (Fig. 6c,d). This local loss of H3K27ac signal occurred at putative enhancers but not at promoters within TACL domains (Fig. 6e and Extended Data Fig. 7a,b). Promoters also did not alter their H3K4me3 levels because of TACL (Extended Data Fig. 7c,d). Switching the TACL system off by the addition of Dox for 1 h did not restore the H3K27ac levels (Fig. 6f,g and Extended Data Fig. 7e). A similarly subtle but significant effect was also seen when analyzing chromatin accessibility by ATAC-seq. Out of ~90,000 identified accessible sites, only 45 and 161 sites showed a significant gain and loss of accessibility, respectively, in TACL versus Cherry cells. Both categories were highly enriched in TACL domains, however, and were not reversible within 1 h of Dox treatment (Fig. 6h,i). Therefore, our data suggest that prolonged exposure to activated cohesin loop extrusion can have an impact on the accessibility and the epigenetic landscape of chromatin, causing mild alterations in H3K27ac levels and the accessibility of potential regulatory sites.

## Discussion

**Extended Data Fig. 8:**
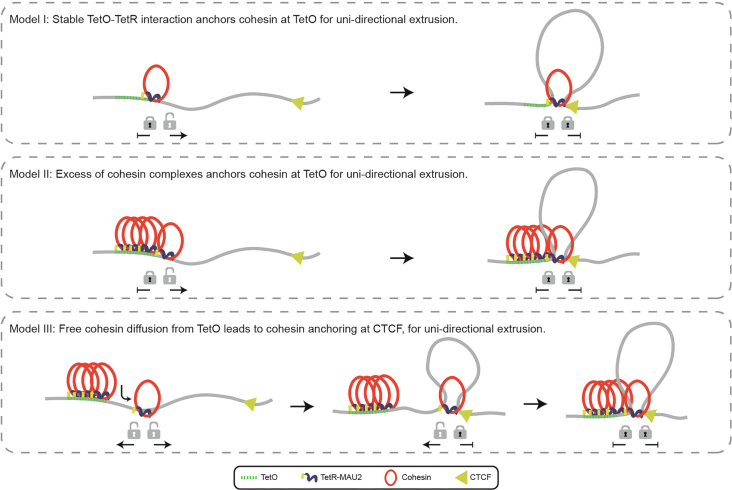
Models of how TACL-recruited cohesin complexes induce single-sided loop extrusion at TetO. Model I assumes anchoring of extruding cohesin at TetO through the stable interaction of TetR-MAU2 with TetO. Model II proposes that the crowding of cohesin complexes at TetO arrays hinders loop extrusion and anchors each individual cohesin complex, such that only an outerbound cohesin complex can perform outward single-sided loop extrusion, Model III assumes that recruited cohesin complexes linearly diffuse away from TetO along DNA until encountering and anchoring at a convergently oriented CTCF site, for uni-directional extrusion that will stall at the crowded TetO sites.

We developed TACL, a system for targeted recruitment of extruding cohesin complexes in living cells. The system appeared to particularly support unidirectional loop extrusion. This may be explained by different, currently indistinguishable, scenarios (Extended Data Fig. 8). The relatively stable TetR–TetO interaction may anchor the entire cohesin complex long enough on DNA to predominantly support single-sided loop extrusion from TetO. More likely, however, the excessive number of cohesin complexes recruited and bound to TetO sites may form a physical barrier for inward-directed loop extrusion. Consequently, only the outer-positioned cohesin molecules independently reel in upstream-flanking or downstream-flanking DNA sequences. A third possibility is that recruited cohesin complexes first migrate away from TetO in an unanchored manner. Upon encountering a convergent CTCF barrier, cohesin will anchor to reel in flanking DNA. TetO, with its densely bound cohesin complexes, could then serve as an impenetrable barrier to halt this extrusion, resulting in temporarily stabilized CTCF–TetO loops.

We found that most CTCF-bound DNA sites have the capacity to halt cohesin^STAG2^ and to form temporarily stabilized chromatin loops with other loop anchors. We propose that such transiently stabilized intradomain CTCF–CTCF interactions are constantly formed throughout the genome, but in wild-type cells, they probably remain undetectable owing to their low frequency across cell populations. Without TACL tremendously boosting loop extrusion from defined anchors, these transient events are too rare among the majority of un-looped alleles to be captured by population-based Hi-C or single-cell Hi-C analysis.

With TACL, we demonstrated that activated cohesin loop extrusion negatively influenced gene expression and also reduced H3K27ac levels of sites in the TACL domains. Although the effect sizes of these changes were small, this result demonstrated that the extruding cohesin machinery can influence the epigenetic makeup of chromatin and the activity of genes that it passes. We currently have no evidence that there is a causal relationship between the two observations. It seems intuitive to interpret the decrease in H3K27ac levels as an indication of reduced local enhancer activity, but we do not expect that all responding genes need enhancers for their expression. Rather, we believe that gene transcription may be hampered by encounters between the RNA polymerase transcription machinery and the cohesin loop extrusion machinery, which both traverse along the chromatin template. We currently do not know how frequent these encounters are: they will not only depend on the density of loop-extruding cohesin complexes on chromatin, but also on gene sizes and their (unknown) frequencies of transcriptional bursts. Enhancers are believed to be natural chromatin loading sites of cohesin. Our finding that cohesin can hamper gene transcription is in line with recent observations that genes proximal to enhancers are upregulated upon depletion of cohesin^36,45,56^. Why some sites lose K27ac when exposed to high cohesin loop extrusion activity remains to be investigated.

An unexpected observation was the TACL-induced, cohesin-dependent accumulation of NIPBL and MAU2 at flanking CTCF sites. This was not a peculiarity of the TACL system, as we also observed this happening elsewhere in the genome, both in TACL and in non-TACL (Cherry) cells, and in datasets published by others^12^. It was most notable in TACL cells, also outside the TACL domains. This could be because of the 1.8-fold elevated nuclear MAU2 levels present in TACL cells. Alternatively, TetR–MAU2 molecules may be better cross-linkable to DNA than normal MAU2, facilitating their detection by ChIP. The strong TACL-induced accumulation of NIPBL–MAU2 at CTCF sites may be the consequence of cohesin complexes queuing behind the CTCF-stalled cohesin complex. Although cohesin complexes directly interacting with CTCF probably have NIPBL–MAU2 displaced by PDS5A^3,29,30^, the upstream and downstream queuing cohesin complexes may still carry NIPBL–MAU2 and hence accumulate these proteins around CTCF sites. Supporting this scenario, NIPBL indeed piles up at some distance on both sides of the CTCF binding sites (Fig. 5b). Our data may also suggest that CTCF-anchored loop formation and stabilization requires sustained active engagement of NIPBL and MAU2.

By enabling spatially and temporally controlled activation of cohesin loop extrusion, TACL opens new avenues for exploring the dynamics of individual loop extrusion trajectories in vivo.

## Methods

Experiments performed in this study did not require ethics board approval.

### Cell culture

HAP1 cells were cultured in Iscove’s modified Dulbecco’s medium (IMDM) supplemented with GlutaMAX (Thermo Fisher Scientific), 25 mM HEPES, 10% FBS and 1% penicillin–streptomycin, following standard procedures. Cells were routinely checked and sorted for haploidy. All 293TX cells were cultured in DMEM supplemented with 10% FBS and 1% penicillin–streptomycin.

### Antibodies

Antobidoes used included Anti-SMC1 (A300-055A, Bethyl), anti-SMC3 (A300-060A, Bethyl), anti-RAD21 (05-908, Merck), anti-NIPBL (A301-779A, Bethyl), anti-FLAG (F1804, Merck), anti-SCC4/MAU2 (ab183033, Abcam), anti-GAPDH (sc-32233, Santa Cruz), anti-STAG1 (A302-579A, Bethyl), anti-STAG2 (A300-159A, Bethyl), anti-CTCF (ab128873, Abcam), anti-H3K4me3 (39060, Active motif), anti-H3K27ac (39133, Active motif), anti-V5 (R960-25, Thermo Fisher Scientific), anti-WAPL (sc-365189, Santa Cruz) and anti-PDS5A (A300-089A, Bethyl).

### Plasmid construction

The plasmids expressing TetR–FLAG–MAU2 and TetR–FLAG–mCherry cassettes were cloned into a lentivirus backbone under the control of the EF1 promoter. TetR, FLAG and MAU2 or mCherry sequences were PCR-amplified with 20 bp overhang for In-Fusion cloning. The final expression cassette comprised EF1-TetR-FLAG-MAU2/mCherry-P2A-Puromycin. To construct the V5–MAU2 plasmid, the TetR–FLAG sequence from the TetR–FLAG–MAU2 construct was removed, and a V5 tag was inserted instead. To enable simultaneous expression of the two MAU2 constructs, the antibiotic selection marker was replaced by blasticidin instead of puromycin. To insert the AID2 tag into the endogenous gene, a single guide RNA (sgRNA) targeting the ORF of the gene was cloned into a vector containing SpCas9–T2A–BFP (Supplementary Table 1). To construct the donor template for AID2 tag insertion, a cassette containing AID2–GFP was cloned between two homology arms of about 1 kb surrounding the sgRNA cut site. Detailed plasmid maps can be found in Supplementary Information.

### Generation of cell lines containing the TetO platforms

The plasmids bearing the TetO platforms and the PiggyBac transposase were originally obtained from L. Giorgetti^39^, validated by Nanopore sequencing with 48× repeats (see Supplementary Table 2 for sequences). In brief, HAP1 cells were trypsinized and resuspended in serum-free IMDM medium. A vector containing the PiggyBac transposase (pBroad3_hyPBase_IRES_tagRFPt) was mixed with a PiggyBac donor vector bearing 30× TetO binding sites and polyethylenimine (PEI; Polysciences) in serum-free IMDM. The DNA mix was incubated at room temperature (20–22 °C) for 10 min, after which the cells and the DNA mix were incubated together for another 10 min. The cells were then plated in a six-well plate. After 24 h, the medium was refreshed. Then, 48–72 h after the transfection, the cells were sorted for a RFP signal, expressing the transposase. Sorted cells were plated in a 15 cm dish and cultured for at least 14 days. Colonies were picked and sub-cultured in 96-well plates. To genotype the clones with a sufficient number of integration sites, cells were lysed in DirectPCR lysis reagent (Viagen). Lystes were subsequently assessed by running qPCR with primers annealing to the transposon sequences. A primer targeting a part of the human *FSIP2* gene was used as the reference among different clones. An estimation of the number of integration sites was calculated as: ${2}^{-({\mathrm{Ct}}_{{\rm{T}}{\rm{e}}{\rm{t}}{\rm{O}}\,{\rm{p}}{\rm{r}}{\rm{i}}{\rm{m}}{\rm{e}}{\rm{r}}}-{\mathrm{Ct}}_{\mathrm{Re}{\rm{f}}{\rm{e}}{\rm{r}}{\rm{e}}{\rm{n}}{\rm{c}}{\rm{e}}})}$. The exact number of integration sites was validated by 4C-seq.

### Lentivirus production and transduction

A total of 4 × 10^6^ 293TX cells were plated in a 10 cm dish 24 h before virus production. Lentiviral vectors were co-transfected with pVSV-G, pMDL RRE and pRSV-REV in serum-free DMEM with PEI (Polysciences). The medium was refreshed 18 h after transfection. The medium containing the virus particles was collected 48 h after transfection by passing through a 0.45 μm filter. For transduction, HAP1 cells were plated in a six-well plate 24 h before transduction. The transduction was performed by adding the virus particles directly onto the cells supplemented with 6 μg ml^−1^ polybrene (Merck). The cells were refreshed 24 h after transduction, and antibiotics (puromycin and blasticidin) were added 48 h after transduction. Cells were selected with antibiotics until the cells in the control plate (without transduction) were completely dead.

### Western blot

Cells were washed in PBS and lysed in RIPA buffer with protease inhibitor (Roche) on ice for 15 min. The cell lysate was further disrupted by sonication with Bioruptor Pico (Diagnode). The cell lysate was cleared by spinning at 1,000*g* for 5 min. The supernatant was incubated with Laemmli buffer and boiled for 10 min. The sample was then loaded on a 4–15% Mini-PROTEAN TGX Precast Protein Gel (Bio-Rad) and run at 100 V for 90 min. Proteins were transferred onto a nitrocellulose or PVDF membrane and incubated with the primary antibody overnight at 4 °C. The membrane was then washed in PBS with 0.25% Tween and incubated with the secondary antibody at room temperature for 1 h. Finally, the membrane was incubated with SuperSignal West Pico PLUS Chemiluminescent Substrate (Thermo Fisher Scientific) for 1 min before being visualized on ImageQuant 800 imager (Amersham).

### Nuclear and cytoplasmic fractionation

In brief, 3 × 10^6^ cells were collected by trypsinization. Cells were washed with PBS, and the cell pellet was resuspended in 100 μl of cytoplasmic extraction buffer (10 mM HEPES, 60 mM KCl, 1 mM EDTA, 0.075% (v/v) NP-40, 1 mM dithiothreitol and 1 mM PMSF, final pH 7.6) and incubated on ice for 3 min. The suspension was spun at 1,500 rpm for 4 min, and the supernatant was kept as the cytoplasmic fraction. The pellet was washed once with cytoplasmic extraction buffer. The cells were pelleted at 1,500 rpm for 4 min and resuspended in 50 μl of nuclear extraction buffer (20 mM Tris Cl, 420 mM NaCl, 1.5 mM MgCl_2_, 0.2 mM EDTA, 1 mM PMSF and 25% (v/v) glycerol, final pH 8.0). The salt concentration was adjusted to 400 mM NaCl, and an additional pellet volume of nuclear extraction buffer was added. The pellet was vortexed and incubated on ice for 10 min. The suspension was spun at max speed for 10 min, and the supernatant was kept as the nuclear fraction.

### ChIP

A total of 100 million cells were crosslinked with 1% formaldehyde for 10 min. Cells were subsequently quenched with 125 mM glycine for 10 min and washed twice with cold PBS. Cells were scraped from culture dishes, and cell pellets were subsequently lysed in LB1 buffer (50 mM HEPES, 140 mM NaCl, 1 mM EDTA, 10% glycerol, 0.5% NP-40, 0.25% Triton X-100), washed in LB2 buffer (10 mM Tris, 200 mM NaCl, 1 mM EDTA, 0.5 mM EGTA) and resuspended in LB3 buffer (10 mM Tris, 100 mM NaCl, 1 mM EDTA, 0.5 mM EGTA, 0.1% sodium deoxycholate, 0.5% *N*-lauroylsarcosine) before sonication. Chromatin was sonicated using Bioruptor Pico (Diagnode) with a setting of 30 s on, 30 s off for eight cycles. Fragmented chromatin was then incubated with 6 µg of antibodies pre-coupled to Dynabeads Protein G beads (Thermo Fisher Scientific) overnight at 4 °C. Bead-bound chromatin was then washed 10× with RIPA buffer (50 mM HEPES, 500 mM LiCl, 1 mM EDTA, 1% NP-40, 0.7% sodium deoxycholate), once with TBS buffer and decrosslinked in elution buffer (50 mM Tris, 10 mM EDTA, 1% SDS) at 65 °C for 18 h. Eluted DNA was then treated with protease K and RNAse A, and subsequently purified with phenol/chloroform/isoamyl alcohol 25:24:1. Purified DNA was either assessed with qPCR (see Supplementary Table 1 for oligonucleotides used) or continued with ChIP–seq next-generation sequencing library preparation. Sequencing libraries were constructed using the NEBnext Ultra II DNA library prep kit (New England Biolabs, NEB) following the manufacturer’s protocol. In brief, DNA was end-repaired and poly-A tailed, ligated to NEBnext adaptors and digested with USER enzyme. Annealed libraries were then purified with AMPure XP beads (Beckman Coulter) and PCR-amplified with indexing primers for 4–12 cycles. Sequencing libraries were checked with Bioanalyzer HS DNA chip (Agilent) and sequenced on the Illumina NextSeq 500 (single-end reads, 75 bp) and NextSeq 2000 platforms (paired-end reads, 50 bp).

### 4C-seq

The 4C template preparation was performed as previously described^57,58^. In brief, ten million cells per sample were crosslinked with 2% formaldehyde, followed by quenching by glycine at a final concentration of 0.125 M. The four-cutter restriction enzyme MboI (NEB) was used for in situ digestion (300 U per ten million cells). Digested DNA fragments were ligated, reverse-crosslinked and subsequently purified through isopropanol and magnetic beads (Macherey–Nagel NucleoMag PCR Beads). The four-cutter restriction enzyme Csp6I (CviQI, Thermo Fisher Scientific, ER0211; 50 U per sample) was used for template trimming. Re-ligated and purified 4C templates were further processed through in vitro Cas9 digestion as described below.

### In vitro Cas9 digestion of 4C templates

To prevent PCR amplification and sequencing of TetO repeats owing to tandem ligation of two or more TetO DpnII fragments in a given 4C circle, an in vitro digestion of 4C templates was performed as previously described^59^ with the following modifications: two sgRNAs were used to target Cas9 into the TetO repeats between viewpoint primers; and pre-incubation of the Cas9 protein and sgRNA template was performed at room temperature. In brief, two sgRNA templates were obtained using the Megashortscript T7 transcription kit (Invitrogen), followed by 4× AMPure XP (Agencourt) purification. Purified Cas9 protein (generated by Hubrecht protein facility) was pre-incubated with the sgRNAs for 30 min at room temperature. The 4C templates were subsequently added to the pre-incubated Cas9–sgRNA complexed for overnight digestion at 37 °C. Cas9 protein was inactivated by incubating at 70 °C for 5 min. The resulting products were purified with 1× AMPure XP and used as a PCR template for TetO-dedicated 4C.

### Nascent RNA sequencing (BrU-seq)

BrU-seq was performed as previously described^60^. Cultured cells were incubated with 2 mM bromouridine (BrU, Merck) for 10 min and subsequently lysed in TRIzol reagent (Thermo Fisher Scientific). RNA was isolated following the manufacturer’s protocol. In brief, lysed cells were mixed with chloroform and centrifuged for 15 min. The aqueous phase was transferred to a new tube and mixed with isopropanol. After centrifugation, the RNA pellet was washed once with 70% ethanol and dissolved in DEPC water. To capture BrU-labeled nascent RNA, 6 µg anti-BrdU antibodies (BD Biosciences) pre-coupled with Dynabeads Protein G beads (Thermo Fisher Scientific) were incubated with the total RNA for 1 h at room temperature. The beads were then washed three times with PBS/0.1% Tween-20/RNaseOUT. To purify the bead-bound RNA, TRIzol reagent was directly added to the beads, and RNA was purified as described above. Next-generation sequencing libraries were generated using the NEBnext Ultra II directional RNA library prep kit (NEB) following the manufacturer’s protocol. In brief, RNA was fragmented to about 200 bp in size. First-strand and second-strand cDNA were synthesized. Double-strand cDNA was then end-repaired, poly-A-tailed, ligated to NEBnext adaptors and digested with USER enzyme. Annealed libraries were then purified with AMPure XP beads (Beckman Coulter) and PCR-amplified with indexing primers for seven cycles. Sequencing libraries were checked with Bioanalyzer HS DNA chip (Agilent) and sequenced on the Illumina NextSeq 2000 platforms (single-end reads, 50 bp).

### Hi-C

Hi-C template preparation was performed as previously described^25^. In brief, ten million cells per sample were crosslinked with 2% formaldehyde, followed by quenching by glycine at a final concentration of 0.2 M. The four-cutter restriction enzyme DpnII (NEB) was used for in situ digestion (400 U per ten million cells). Digested DNA was repaired with biotin-14–dATP (Life Technologies) in a Klenow end-filling reaction. End-repaired, ligated and reverse-crosslinked DNA was subsequently purified using isopropanol and magnetic beads (Macherey–Nagel NucleoMag PCR Beads). Purified DNA was sheared to 300–500 bp with Covaris and subsequently size-selected by AMPure XP (Agencourt). Appropriately sized ligation fragments marked by biotin were pulled down with MyOne Streptavidin C1 DynaBeads (Invitrogen) and prepped for Illumina sequencing.

### ATAC-seq

ATAC-seq was conducted following the Omni-ATAC protocol. In summary, 200,000 cells were lysed using a solution containing 0.1% NP-40, 0.1% Tween-20 and 0.01% digitonin, then incubated with a homemade Tagment DNA Enzyme for 30 min at 37 °C. DNA purification was carried out using the QIAGEN MinElute Reaction Cleanup Kit. Library fragments were amplified with Phusion High-Fidelity PCR Master Mix with HF Buffer (Thermo Fisher Scientific, cat. no. F531S) and custom primers featuring unique single or dual indexes. Purification of the libraries was performed using AMPure XP beads (Beckman Coulter, cat. no. A63881), following the manufacturer’s guidelines. The quality of the constructed libraries was assessed using the Agilent Bioanalyzer 2100 with the DNA 7500 kit (cat. no. 5067-1504).

### Generation of auxin-inducible degron cells

To deplete the cohesin factors in cells, we used the AID2 system^49^. For RAD21 degron, we generated HAP1 cells stably expressing OsTIR1 (F74G) by transducing the cells with lentivirus containing an expression cassette of OSTIR1-P2A-hygromycin. After antibiotic selection with hygromycin, cells were co-transfected with a vector expressing an sgRNA against RAD21 and SpCas9–T2A–BFP, and the donor template containing AID-GFP flanked by homology arms. GFP^+^ cells were analyzed and sorted with flow cytometry. Single-cell clones were expanded and used for downstream analysis. For WAPL, PDS5A, STAG2 and CTCF degrons, we first inserted the AID–GFP cassette by co-transfecting the cells with an sgRNA against each gene. Single-cell clones were selected and verified by PCR. Verified clones were then transduced with lentivirus containing an expression cassette of OSTIR1-P2A-blasticidin. To deplete the proteins, we treated the cells with 1 μM auxin (IAA; BioAcademia) for 2–3 h and analyzed the successful depletion with western blot.

### Data analysis

#### 4C-seq

4C-seq reads were mapped to the hg38 reference genome and processed using pipe4C^57^ (https://github.com/deLaatLab/pipe4C). Normalized 4C coverage was calculated separately for each TetO integration site using R (www.r-project.org). Counts at non-blind fragments within a 20 Mb region (10 Mb upstream and downstream of the viewpoint) were adjusted to one million mapped reads after exclusion of the two highest-count fragments. Count data was smoothed using a running mean with a window size of 21 fragments using the R package caTools (v.1.18.2).

##### Aggregate 4C analysis

In 3C-based assays, ligation frequencies are typically highest near the viewpoint (<100 kb) and decrease as the distance from the viewpoint increases. To minimize the high background ligation frequencies close to the viewpoint, only peaks located at least 100 kb away from the TetO integration sites were included in the aggregate 4C analysis. These peaks were resized to 100 kb, divided into 2 kb bins and the average normalized 4C signal was calculated for each bin.

##### TACL domains annotation

To systematically annotate the TACL domains induced by the recruitment of cohesin to the TetO platforms, we developed an HMM. The HMM was implemented using the Python package hmmlearn (https://github.com/hmmlearn/hmmlearn). We created an HMM with the states ‘TACL_domain’ and ‘no_change’. The normalized 4C-seq signals for TACL-ON and Cherry conditions were binarized into two observations: ‘TACL_domain’ (4C-seq signal difference between TACL-ON and Cherry >25) and ‘no_change’ (4C-seq signal difference ≤25). The emission probabilities were estimated using manually defined TACL domains. The probability of the TACL_domain state was calculated as the fraction of restriction fragments with 4C-seq signal >25 in the manually defined TACL domains and set to 0.6. The probability of the no_change state was calculated as the fraction of restriction fragments with 4C-seq signal ≤25 in the flanking regions of the manually defined TACL domains and was set to 0.98. The transition probability was set to 10^−6^.

The estimated TACL_domain and no_change states were then subjected to several additional filters. First, restriction fragments belonging to stretches of more than 20 consecutive TACL_domain states were retained. Second, restriction fragments with consecutive TACL_domain states within 100 kb of each other were merged. Third, merged regions containing at least 40 restriction fragments were retained and further merged within 1.5 Mb of each other to draft TACL domains. Finally, if TetO was outside of the drafted TACL domain, the closest domain segment on the other side of the domain with respect to the TetO location was added to obtain TACL domains.

HMM model with the same parameters was used to annotate TACL domains in the CTCF–AID, WAPL–AID, STAG2–AID and PDS5A–AID lines by comparing the difference between IAA and Dox treatments. Additionally, the same HMM model was used to annotate STAG2 collapsed domains by comparing the difference between the IAA treatment and the untreated condition in the STAG2–AID line. For the filtering steps, the distance for considering restriction fragments with consecutive TACL_domain states was set to 200 kb, and the distance for drafting TACL domains from restriction fragments was set to 2.5 Mb.

#### ChIP–seq

HAP1 H3K4me1 data are publicly available (ENCODE: ENCSR450JTP).

ChIP–seq reads were mapped to the hg38 reference genome and processed using the 4DN ChIP–seq pipeline (https://github.com/4dn-dcic/chip-seq-pipeline2). *P* value signal bigwigs were used for all heatmaps and example plots. For wild-type, T-MAU2, T-MAU2 treated with Dox or T-mCherry cells, the *P* value signals were normalized based on the average *P* value signal for all CTCF peaks in TACL-ON (for CTCF, RAD21, SMC1, SMC3, STAG1, STAG2, WAPL, PDS5A), FLAG peaks in TACL-ON (for FLAG, MAU2, NIPBL, V5), H3K27ac peaks (H3K27ac) or H3K4me3 peaks (H3K4me3) located outside the TACL domains and further than 3 Mb from the TetO integration sites. In brief, ChIP–seq peaks were filtered for a ‘signalValue’ that represented clear peaks by visual inspection (CTCF, 35; FLAG–MAU2, 35) and for overlapping peaks, such that for overlapping peaks the peak with the highest signalValue was kept. Filtered peaks were resized to 10 bp, and the signal was calculated using the GenomicRanges and rtracklayer package in R/Bioconductor^61,62^. The average signal was used as the scaling factor. For degron lines, *P* value signals were normalized based on the average signal of the regions flanking the filtered peaks. In brief, peaks were filtered for signalValue and for overlapping peaks as described above. Next, peaks were resized to 5 kb, and the signals of the outer 1 kb regions (2.5–1.5 kb upstream and downstream of the peak center) were calculated. The average signal of the outer 1 kb regions was used as the scaling factor. For heatmaps, the signal coverage was calculated per 10 bp bin as described above and normalized using the previously determined scaling factor. For the average ChIP signal plot, the average signal for each 10 bp bin was calculated. TetO enrichment ChIP–seq reads were mapped to the hg38 human reference genome assembly with added minimal PiggyBac TetO sequence (Supplementary Table 2) using bowtie2 (v.2.5.2)^63^. Alignments with a mapping quality (MAPQ) score of ≥1, either to the PiggyBac TetO sequence or elsewhere in the genome, were quantified using FeatureCounts (v.2.0.6)^64^. Enrichment levels were determined by comparing the coverage to the average coverage from all input control experiments.

##### Differential FLAG peaks

FLAG ChIP–seq reads were aligned as single-end reads to the hg38 human reference genome assembly with added TetO sequence using bowtie2 (v.2.5.2)^63^. Reads with MAPQ ≥ 15 were selected using SAMtools (v.1.15)^65^, and duplicate reads were removed with the Picard (v.2.25.6) ‘MarkDuplicates’ function (https://broadinstitute.github.io/picard). Coverage over FLAG peaks was then quantified using FeatureCounts (v.2.0.6)^64^ and normalized with DESeq2 (v.1.38.3)^66^. For TACL‑ON samples, an average signal was calculated by taking the mean of the two replicates. With the addition of a pseudocount of 1, the log_2_(fold change) between TACL‑ON and TACL‑OFF conditions was computed. Differential FLAG peaks were defined as those with a log_2_(fold change) value of >1 and with an average TACL‑ON signal exceeding 2^4.5^.

##### Classification of CTCF sites

Genome-wide CTCF sites were defined as those CTCF peaks located outside of TACL domains and at least 3 Mb away from any TetO integration site in TACL-ON cells. To stratify these sites by CTCF binding strength, we used the ChIP–seq coverage values in TACL-ON. Peaks with a signal below the 33^rd^ quantile were classified as low, those between the 33^rd^ and 66^th^ quantiles as medium and those above the 66^th^ quantile as high.

The presence and orientation of CTCF motifs below each CTCF peak were identified using FIMO (v.5.3.0)^67^ using the MA0139.1 motif^68^ and the parameter --max-stored-scores 50,000,000. CTCF peaks for which all identified motifs were located on the plus strand were classified as forward CTCF peaks, while peaks for which all identified motifs were located on the minus strand were classified as reverse CTCF peaks. Forward CTCF motifs located upstream of TetO sites and reverse CTCF motifs located downstream of CTCF were classified as convergent CTCF binding sites. Reverse CTCF motifs located upstream of TetO sites and forward CTCF motifs located downstream of CTCF were classified as divergent CTCF binding sites.

#### Analysis of ATAC-seq and ChIP–seq for histone modifications

##### Data processing

ATAC-seq reads were mapped to the hg38 human reference genome assembly using bwa mem (v.0.7.17-r1188)^69^. ChIP–seq reads were mapped to the hg38 human reference genome assembly with added TetO sequence using bowtie2 (v.2.5.2)^63^. Uniquely mapped reads in proper read pairs (-f 2) with MAPQ > 10 and MAPQ ≥ 15 were selected using SAMtools (v.1.15)^65^ for ATAC-seq and ChIP–seq data, respectively. Duplicate reads were filtered out using the Picard (v.2.25.6) and (v.3.1.1) ‘MarkDuplicates’ function (https://broadinstitute.github.io/picard) for ATAC-seq and ChIP–seq data, respectively. Bigwig coverage tracks were generated using the ‘bamCoverage’ function from the deepTools (v.3.4.2)^70^ with the ‘–effectiveGenomeSize’ parameter set to 2,913,022,398 and ‘–normalizeUsing’ parameter set to RPGC.

##### Peak calling

Peaks were called using MACS2 (v.2.2.6)^71^ for pooled data and replicates in a narrowPeak mode, with mappable genome size set to hs, a *q* value cutoff of 0.05, ‘–keep-dup’ parameter set to all and the ‘–nomodel’ parameter. The consensus peak list was obtained by overlapping the peaks called for pooled data with peaks from replicates. Only the peaks from canonical chromosomes outside of the blacklist regions^72^ that had an overlap of at least 50% with peaks from both replicates were retained.

##### Peak analysis

ATAC-seq and H3K27ac peaks from the TACL-ON, TACL-OFF and Cherry conditions were pooled into one set for differential occupancy analysis. Peak counts were obtained using the ‘intersect’ function from BEDTools (v.2.27.1)^73^ with ‘-c -wa’ parameters. Differential ATAC-seq and H3K27ac peaks were identified using the DESeq2 (v.1.30.1)^66^. The ‘nbinomWaldTest’ function with default parameters was used to test contrasts. Peaks with a false discovery rate of <0.05 and an absolute log_2_(fold change) of >0.5 were considered significant. For downstream analyses, peak overlap was performed using Bioframe (v.0.3.0). H3K27ac peaks that overlapped with H3K4me3 peaks were classified as promoter peaks, and non-overlapping peaks were classified as enhancer peaks.

#### Bru-seq

##### Data processing

BrU-seq reads were mapped to the hg38 human reference genome assembly using STAR (v.2.7.9a)^74^ with GENCODE (v.44) gene annotation. Uniquely mapped reads with MAPQ > 10 were selected and split by strand using SAMtools (v.1.12)^65^. Forward strand reads were extracted by using -f 16 FLAG, and reverse strand reads were extracted by using -F 16 FLAG. Gene counts were obtained using the ‘htseq-count’ function from HTSeq (v.0.13.5)^75^. Counts were calculated separately for genes from forward and reverse strands with the parameters ‘–stranded no’, ‘–nonunique all’, ‘–order pos’ and ‘–type gene’.

##### Differential expression analysis

Differentially expressed genes were identified using the DESeq2 (v.1.30.1)^66^ (Supplementary Table 3). Low-expressed genes were filtered by requiring the samples to have gene counts greater than ten. The ‘nbinomWaldTest’ function with default parameters was used to test contrasts. Genes with a false discovery rate of <0.05 and an absolute log_2_(fold change) of >1 were considered significant. For downstream analyses, the genes were overlapped with annotated TACL domains and split into groups depending on their relative distance and position to the TetO platforms using bioframe (v.0.3.0).

#### Hi-C analysis

##### Data processing

Hi-C data was processed using the distiller pipeline from Open2C (https://github.com/open2c/distiller-nf). The reads were mapped to the human reference genome assembly hg38 with bwa mem (v.0.7.17-r1188)^69^ with ‘-SP’ FLAGs. The alignments were parsed and filtered for duplicates using the pairtools (v.0.3.0)^76^. The complex walks in long reads were masked with ‘–walks-policy’ set to mask, the maximal allowed mismatch for reads to be considered as duplicates ‘max_mismatch_bp’ was set to 1, and the mapping quality threshold was set to 30. Filtered read pairs were aggregated into genomic bins of different sizes using the cooler (v.0.8.11)^77^. The resulting Hi-C matrices were normalized using the iterative correction procedure.

##### Compartment annotation

A and B compartments were annotated using the cooltools (v.0.3.2) call-compartments function for 200 kb resolution contact matrices. The orientation of the eigenvectors (PC1) was selected such that it correlates positively with GC content and expression data. Consequently, B compartment bins were assigned with negative eigenvector values, and A compartment bins were assigned with positive.

##### Loops and TADs annotation

High-resolution Hi-C data for HAP1 cells^22^ at 10 kb resolution were used for loops and TADs annotation. Loops were annotated using Chromosight (v.1.4.1)^78^. For loop detection, the Pearson correlation threshold was set to 0.4, loop sizes were set between 50 kb and 5 Mb and the parameter ‘–smooth-trend’ was enabled. TADs were annotated using the insulation score algorithm implemented in the cooltools (v.0.3.2) diamond-insulation function^79^. The window size for insulation score calculations was set to 200 kb. The threshold for the boundary strength filter was calculated using the Li method, implemented in the scikit-image package^80^. The bins with boundary strength higher than ~0.19 were considered as TAD boundary bins. These bins were converted into TADs by continuously joining two neighboring bins together. The TAD boundary coordinate was then randomly selected from the coordinates of the joined bins with a significant insulation score.

##### Aggregate analyses

Average loops, TAD boundaries and TADs were calculated for 10 kb resolution observed-over-expected Hi-C contact matrices using the loops and TADs annotated as described above. Publicly available HAP1 Hi-C data were included for comparison^6,22^. Expected contact matrices were obtained using the cooltools (v.0.3.2) function ‘compute-expected’^79^. Average loops were generated using coolpup.py (v.0.9.5)^81^ with ‘pad’ set to 200 and ‘min-dist’ set to 0. Average TAD boundaries were generated using coolpup.py (v.0.9.5)^81^ in ‘local’ mode with ‘pad’ set to 500. Average TADs generated using coolpup.py (v.0.9.5)^81^ in ‘local’ mode with the ‘rescale’ option, with the ‘rescale_size’ set to 99. The average loop strength was calculated as the mean value of the central three-by-three square pixels. The average TAD boundary strength was calculated as the mean value of the average intra-TAD interactions (upper-left and bottom-right quarters) divided by the mean value of average inter-TAD interactions (upper-right and bottom-left quarters). The average TAD density was calculated as the mean value of the central 33-by-33 square pixels.

The aggregate stripes analysis of the TetO integrations was performed using cooltools (v.0.5.1)^79^ and bioframe (v.0.3.0)^82^ for 10 kb resolution observed-over-expected Hi-C contact matrices. The pile-ups of the TetO integrations were created using the cooltools.pileup function with 500 kb regions around the integration coordinates as flanks.

### Statistics and reproducibility

All comparisons were made between biologically independent samples. No statistical method was used to predetermine sample size. No data were excluded from the analyses. The experiments were not randomized. The Investigators were not blinded to allocation during experiments and outcome assessment. Data distribution was assumed to be normal, but this was not formally tested.

### Reporting summary

Further information on research design is available in the Nature Portfolio Reporting Summary linked to this article.

## Online content

Any methods, additional references, Nature Portfolio reporting summaries, source data, extended data, supplementary information, acknowledgements, peer review information; details of author contributions and competing interests; and statements of data and code availability are available at 10.1038/s41588-025-02358-0.

## Supplementary information

Reporting Summary

Supplementary Table 1 Oligo list.

Supplementary Table 2 TetO sequences.

Supplementary Table 3 Sequencing read count table.

Supplementary Data 1 1_PB-empty_TetO.gb. 2_Lenti-TetR-mcherry.gb. 3_Lenti-TetR-Mau2-puro.gb. 4_Cas9-2A-BFP.gb. 5_Rad21-AID-GFP-Blast.gb. 6_CTCF-AID-GFP.gb. 7_PDS5A-AID-GFP.gb. 8_STAG2-AID-GFP.gb. 9_WAPL-AID-GFP.gb.

## Source data

Source Data Fig. 4 Unprocessed western blots.

Source Data Extended Data Fig. 1 Unprocessed western blots.

Source Data Extended Data Fig. 4 Unprocessed western blots.

Source Data Extended Data Fig. 5 Unprocessed western blots.

## Data Availability

Processed sequencing data for this study are available at the NCBI Gene Expression Omnibus under accession GSE218803. Source data are provided with this paper.

## Code availability

Code used for data analysis is available via Zenodo at 10.5281/zenodo.15777998 (ref. ^83^) and 10.5281/zenodo.15782876 (ref. ^84^).
