# Supplementary material for: Characterization of induced cohesin loop extrusion trajectories in living cells
Source: Nat Genet. 2025 Oct 16;57(11):2785–97. doi: 10.1038/s41588-025-02358-0 (PMC12597828; doi:10.1038/s41588-025-02358-0)
Supplement: Supplementary file 1 — Reporting Summary [file 41588_2025_2358_MOESM1_ESM.pdf]

nature portfolio

Corresponding author(s): Ruiqi Han
Wouter de Laat

Last updated by author(s): Jun 26, 2025

Reporting Summary

Nature Portfolio wishes to improve the reproducibility of the work that we publish. This form provides structure for consistency and transparency in reporting. For further information on Nature Portfolio policies, see our Editorial Policies and the Editorial Policy Checklist.

Statistics

For all statistical analyses, confirm that the following items are present in the figure legend, table legend, main text, or Methods section.

n/a	Confirmed	
☐	☒	The exact sample size (*n*) for each experimental group/condition, given as a discrete number and unit of measurement
☐	☒	A statement on whether measurements were taken from distinct samples or whether the same sample was measured repeatedly
☐	☒	The statistical test(s) used AND whether they are one- or two-sided<br>*Only common tests should be described solely by name; describe more complex techniques in the Methods section.*
☐	☒	A description of all covariates tested
☒	☐	A description of any assumptions or corrections, such as tests of normality and adjustment for multiple comparisons
☐	☒	A full description of the statistical parameters including central tendency (e.g. means) or other basic estimates (e.g. regression coefficient) AND variation (e.g. standard deviation) or associated estimates of uncertainty (e.g. confidence intervals)
☐	☒	For null hypothesis testing, the test statistic (e.g. *F*, *t*, *r*) with confidence intervals, effect sizes, degrees of freedom and *P* value noted<br>*Give P values as exact values whenever suitable.*
☒	☐	For Bayesian analysis, information on the choice of priors and Markov chain Monte Carlo settings
☒	☐	For hierarchical and complex designs, identification of the appropriate level for tests and full reporting of outcomes
☒	☐	Estimates of effect sizes (e.g. Cohen's *d*, Pearson's *r*), indicating how they were calculated

Our web collection on statistics for biologists contains articles on many of the points above.

Software and code

Policy information about availability of computer code

Data collection	Biorad CFX Maestro 2.3, Flowjo v10
Data analysis	4C-seq was analyzed with Pipe 4C (https://github.com/deLaatLab/pipe4C), ChIP-seq was analyzed with 4DN ChIP-seq pipeline (https://github.com/4dn-dcic/chip-seq-pipeline2), BrU-seq was analyzed with DESeq2 (http://bioconductor.org/packages/release/bioc/html/DESeq2.html), Hi-C was analyzed with Open2C (https://open2c.github.io/), customized codes can be found at https://doi.org/10.5281/zenodo.15782875 and https://doi.org/10.5281/zenodo.15777997.

For manuscripts utilizing custom algorithms or software that are central to the research but not yet described in published literature, software must be made available to editors and reviewers. We strongly encourage code deposition in a community repository (e.g. GitHub). See the Nature Portfolio guidelines for submitting code & software for further information.

Data

Policy information about availability of data

All manuscripts must include a data availability statement. This statement should provide the following information, where applicable:

- Accession codes, unique identifiers, or web links for publicly available datasets
- A description of any restrictions on data availability
- For clinical datasets or third party data, please ensure that the statement adheres to our policy

Cell lines, plasmids, and other materials are available upon request. Sequencing data are available on Gene Expression Omnibus database under accession number GSE218803.

Research involving human participants, their data, or biological material

Policy information about studies with human participants or human data. See also policy information about sex, gender (identity/presentation), and sexual orientation and race, ethnicity and racism.

Reporting on sex and gender	n/a
Reporting on race, ethnicity, or other socially relevant groupings	n/a
Population characteristics	n/a
Recruitment	n/a
Ethics oversight	n/a

Note that full information on the approval of the study protocol must also be provided in the manuscript.

Field-specific reporting

Please select the one below that is the best fit for your research. If you are not sure, read the appropriate sections before making your selection.

☒ Life sciences ☐ Behavioural & social sciences ☐ Ecological, evolutionary & environmental sciences

For a reference copy of the document with all sections, see nature.com/documents/nr-reporting-summary-flat.pdf

Life sciences study design

All studies must disclose on these points even when the disclosure is negative.

Sample size	Sample size for each experiment is indicated in the legend. No statistical method was used to predetermine sample size
Data exclusions	No data were excluded from the analyses.
Replication	For ChIP-seq and ATAC-seq, all experiments were repeated at least twice. For Bru-seq, T-mCherry_dox experiments were performed once and T-MAU2 experiments were repeated three times. For Hi-C experiments, the samples indicated were performed once.
Randomization	The experiments were not randomized
Blinding	The investigators were not blinded to allocation during experiments and outcome assessment.

Reporting for specific materials, systems and methods

We require information from authors about some types of materials, experimental systems and methods used in many studies. Here, indicate whether each material, system or method listed is relevant to your study. If you are not sure if a list item applies to your research, read the appropriate section before selecting a response.

Materials & experimental systems

n/a	Involved in the study
☐	☒ Antibodies
☐	☒ Eukaryotic cell lines
☒	☐ Palaeontology and archaeology
☒	☐ Animals and other organisms
☒	☐ Clinical data
☒	☐ Dual use research of concern
☒	☐ Plants

Methods

n/a	Involved in the study
☐	☒ ChIP-seq
☒	☐ Flow cytometry
☒	☐ MRI-based neuroimaging

Antibodies

Antibodies used	Anti-SMC1 (A300-055A, Bethyl), anti-SMC3 (A300-060A, Bethyl), anti-RAD21 (05-908, Merck), anti-NIPBL (A301-779A, Bethyl), anti-FLAG (F1804, Merck), anti-SCC4/MAU2 (ab183033, Abcam), anti-GAPDH (sc-32233, Santa Cruz), anti-STAG1 (A302-579A, Bethyl), anti-STAG2 (A300-159A, Bethyl), anti-CTCF (ab128873, Abcam), anti-H3K4me3 (39060, Active motif), anti-H3K27ac (39133, Active motif), anti-V5 (R960-25, Thermo fisher), anti-WAPL (sc-365189, Santa Cruz), anti-PDS5A (A300-089A, Bethyl)
Validation	Antibody validation can be found on manufacturers' websites.

Eukaryotic cell lines

Policy information about cell lines and Sex and Gender in Research

Cell line source(s)	Human haploid chronic myelogenous leukemia HAP1 cells were used in this study. (not authenticated cell line, available in our institute, periodically tested for mycoplasma)
Authentication	n/a
Mycoplasma contamination	HAP1 cells were periodically tested for mycoplasma.
Commonly misidentified lines (See ICLAC register)	n/a

Plants

Seed stocks	n/a
Novel plant genotypes	n/a
Authentication	n/a

ChIP-seq

Data deposition

☒ Confirm that both raw and final processed data have been deposited in a public database such as GEO.

☒ Confirm that you have deposited or provided access to graph files (e.g. BED files) for the called peaks.

Data access links *May remain private before publication.*	https://www.ncbi.nlm.nih.gov/geo/query/acc.cgi?acc=GSE218803
Files in database submission	GSM8526139 C17,TACL-ON,CTCF,rep1<br>GSM8526140 C17,TACL-ON,FLAG,rep1<br>GSM8526141 C17,TACL-ON,FLAG,rep2<br>GSM8526142 C17,TACL-ON,H3K27ac,rep1<br>GSM8526143 C17,TACL-ON,H3K27ac,rep2<br>GSM8526144 C17,TACL-ON,H3K4me3,rep1<br>GSM8526145 C17,TACL-ON,H3K4me3,rep2

GSM8526146 C17,TACL-ON,Input,rep1
GSM8526147 C17,TACL-ON,Input,rep2
GSM8526148 C17,TACL-ON,Input,rep3
GSM8526149 C17,TACL-ON,Input,rep4
GSM8526150 C17,TACL-ON,Input,rep5
GSM8526151 C17,TACL-ON,Input,rep6
GSM8526152 C17,TACL-ON,Input,rep7
GSM8526153 C17,TACL-ON,MAU2,rep1
GSM8526154 C17,TACL-ON,MAU2,rep2
GSM8526155 C17,TACL-ON,NIPBL,rep2
GSM8526156 C17,TACL-ON,PDS5A,rep1
GSM8526157 C17,TACL-ON,RAD21,rep1
GSM8526158 C17,TACL-ON,RAD21,rep3
GSM8526159 C17,TACL-ON,SMC1,rep2
GSM8526160 C17,TACL-ON,SMC1,rep3
GSM8526161 C17,TACL-ON,STAG1,rep1
GSM8526162 C17,TACL-ON,STAG2,rep1
GSM8526163 C17,TACL-ON,WAPL,rep1
GSM8526164 C17,TACL-OFF,FLAG,rep2
GSM8526165 C17,TACL-OFF,H3K27ac,rep1
GSM8526166 C17,TACL-OFF,H3K27ac,rep2
GSM8526167 C17,TACL-OFF,H3K4me3,rep1
GSM8526168 C17,TACL-OFF,H3K4me3,rep2
GSM8526169 C17,TACL-OFF,Input,rep1
GSM8526170 C17,TACL-OFF,Input,rep2
GSM8526171 C17,TACL-OFF,Input,rep3
GSM8526172 C17,TACL-OFF,MAU2,rep2
GSM8526173 C17,TACL-OFF,NIPBL,rep2
GSM8526174 C17,TACL-OFF,RAD21,rep3
GSM8526175 C17,TACL-OFF,SMC1,rep2
GSM8526176 C17,TACL-OFF,SMC1,rep3
GSM8526177 C17,TACL-OFF,STAG1,rep1
GSM8526178 C17,TACL-OFF,STAG2,rep1
GSM8526179 C17,mCherry,CTCF,rep1
GSM8526180 C17,mCherry,FLAG,rep1
GSM8526181 C17,mCherry,H3K27ac,rep1
GSM8526182 C17,mCherry,H3K27ac,rep2
GSM8526183 C17,mCherry,H3K4me3,rep1
GSM8526184 C17,mCherry,H3K4me3,rep2
GSM8526185 C17,mCherry,Input,rep1
GSM8526186 C17,mCherry,Input,rep2
GSM8526187 C17,mCherry,Input,rep3
GSM8526188 C17,mCherry,Input,rep4
GSM8526189 C17,mCherry,Input,rep5
GSM8526190 C17,mCherry,Input,rep6
GSM8526191 C17,mCherry,MAU2,rep1
GSM8526192 C17,mCherry,MAU2,rep2
GSM8526193 C17,mCherry,NIPBL,rep1
GSM8526194 C17,mCherry,NIPBL,rep2
GSM8526195 C17,mCherry,PDS5A,rep1
GSM8526196 C17,mCherry,RAD21,rep1
GSM8526197 C17,mCherry,RAD21,rep3
GSM8526198 C17,mCherry,SMC1,rep2
GSM8526199 C17,mCherry,SMC1,rep3
GSM8526200 C17,mCherry,STAG1,rep1
GSM8526201 C17,mCherry,STAG2,rep1
GSM8526202 C17,mCherry,WAPL,rep1
GSM8526203 C17,TACL-ON_CRISPRi-Ctrl,Input,rep1
GSM8526204 C17,TACL-ON_CRISPRi-WAPL,Input,rep1
GSM8526205 C17_CTCFAID_C22,TACL-ON,FLAG,rep1
GSM8526206 C17_CTCFAID_C22,TACL-ON,FLAG,rep2
GSM8526207 C17_CTCFAID_C22,TACL-ON,Input,rep1
GSM8526208 C17_CTCFAID_C22,TACL-OFF,FLAG,rep1
GSM8526209 C17_CTCFAID_C22,TACL-OFF,FLAG,rep2
GSM8526210 C17_CTCFAID_C22,TACL-ON_IAA,FLAG,rep1
GSM8526211 C17_CTCFAID_C22,TACL-ON_IAA,FLAG,rep2
GSM8526212 C17_CTCFAID_C22,TACL-OFF_IAA,FLAG,rep1
GSM8526213 C17_CTCFAID_C22,TACL-OFF_IAA,FLAG,rep2
GSM8526214 C17_PDS5AAID_B10,TACL-ON,FLAG,rep1
GSM8526215 C17_PDS5AAID_B10,TACL-ON,FLAG,rep2
GSM8526216 C17_PDS5AAID_B10,TACL-ON,Input,rep1
GSM8526217 C17_PDS5AAID_B10,TACL-ON,NIPBL,rep1
GSM8526218 C17_PDS5AAID_B10,TACL-OFF,FLAG,rep1
GSM8526219 C17_PDS5AAID_B10,TACL-OFF,FLAG,rep2
GSM8526220 C17_PDS5AAID_B10,TACL-OFF,NIPBL,rep1
GSM8526221 C17_PDS5AAID_B10,TACL-ON_IAA,FLAG,rep1

GSM8526222 C17_PDS5AAID_B10,TACL-ON_IAA,FLAG,rep2
GSM8526223 C17_PDS5AAID_B10,TACL-ON_IAA,NIPBL,rep1
GSM8526224 C17_RAD21AID_D8,mCherry,MAU2,rep1
GSM8526225 C17_RAD21AID_D8,mCherry_IAA,MAU2,rep1
GSM8526226 C17_RAD21AID_SC1,TACL-ON,FLAG,rep1
GSM8526227 C17_RAD21AID_SC1,TACL-ON,Input,rep1
GSM8526228 C17_RAD21AID_SC1,TACL-ON,Input,rep2
GSM8526229 C17_RAD21AID_SC1,TACL-ON,Input,rep3
GSM8526230 C17_RAD21AID_SC1,TACL-ON,MAU2,rep1
GSM8526231 C17_RAD21AID_SC1,TACL-ON,NIPBL,rep1
GSM8526232 C17_RAD21AID_SC1,TACL-ON,RAD21,rep1
GSM8526233 C17_RAD21AID_SC1,TACL-ON,SMC1,rep1
GSM8526234 C17_RAD21AID_SC1,TACL-ON,SMC1,rep2
GSM8526235 C17_RAD21AID_SC1,TACL-ON,SMC3,rep1
GSM8526236 C17_RAD21AID_SC1,TACL-ON_IAA,FLAG,rep1
GSM8526237 C17_RAD21AID_SC1,TACL-ON_IAA,Input,rep1
GSM8526238 C17_RAD21AID_SC1,TACL-ON_IAA,Input,rep2
GSM8526239 C17_RAD21AID_SC1,TACL-ON_IAA,MAU2,rep1
GSM8526240 C17_RAD21AID_SC1,TACL-ON_IAA,NIPBL,rep1
GSM8526241 C17_RAD21AID_SC1,TACL-ON_IAA,RAD21,rep1
GSM8526242 C17_RAD21AID_SC1,TACL-ON_IAA,SMC1,rep1
GSM8526243 C17_RAD21AID_SC1,TACL-ON_IAA,SMC1,rep2
GSM8526244 C17_RAD21AID_SC1,TACL-ON_IAA,SMC3,rep1
GSM8526245 C17_RAD21AID_SC6,mCherry,Input,rep1
GSM8526246 C17_RAD21AID_SC6,mCherry,Input,rep2
GSM8526247 C17_RAD21AID_SC6,mCherry,Input,rep3
GSM8526248 C17_RAD21AID_SC6,mCherry,NIPBL,rep1
GSM8526249 C17_RAD21AID_SC6,mCherry,RAD21,rep1
GSM8526250 C17_RAD21AID_SC6,mCherry,SMC1,rep1
GSM8526251 C17_RAD21AID_SC6,mCherry,SMC1,rep2
GSM8526252 C17_RAD21AID_SC6,mCherry_IAA,Input,rep1
GSM8526253 C17_RAD21AID_SC6,mCherry_IAA,Input,rep2
GSM8526254 C17_RAD21AID_SC6,mCherry_IAA,NIPBL,rep1
GSM8526255 C17_RAD21AID_SC6,mCherry_IAA,RAD21,rep1
GSM8526256 C17_RAD21AID_SC6,mCherry_IAA,SMC1,rep1
GSM8526257 C17_STAG2AID_C2,TACL-ON,FLAG,rep1
GSM8526258 C17_STAG2AID_C2,TACL-ON,FLAG,rep2
GSM8526259 C17_STAG2AID_C2,TACL-ON,Input,rep1
GSM8526260 C17_STAG2AID_C2,TACL-ON,STAG1,rep1
GSM8526261 C17_STAG2AID_C2,TACL-OFF,FLAG,rep1
GSM8526262 C17_STAG2AID_C2,TACL-OFF,FLAG,rep2
GSM8526263 C17_STAG2AID_C2,TACL-OFF,STAG1,rep1
GSM8526264 C17_STAG2AID_C2,TACL-ON_IAA,FLAG,rep1
GSM8526265 C17_STAG2AID_C2,TACL-ON_IAA,FLAG,rep2
GSM8526266 C17_STAG2AID_C2,TACL-ON_IAA,NIPBL,rep1
GSM8526267 C17_STAG2AID_C2,TACL-ON_IAA,SMC1,rep1
GSM8526268 C17_STAG2AID_C2,TACL-ON_IAA,STAG1,rep1
GSM8526269 C17_V5-MAU2,TACL-ON,Input,rep1
GSM8526270 C17_V5-MAU2,TACL-ON,NIPBL,rep1
GSM8526271 C17_V5-MAU2,TACL-ON,V5,rep1
GSM8526272 C17_V5-MAU2,mCherry,Input,rep1
GSM8526273 C17_V5-MAU2,mCherry,NIPBL,rep1
GSM8526274 C17_V5-MAU2,mCherry,V5,rep1
GSM8526275 C17_WAPLAID_C8,TACL-ON,FLAG,rep1
GSM8526276 C17_WAPLAID_C8,TACL-ON,FLAG,rep2
GSM8526277 C17_WAPLAID_C8,TACL-ON,Input,rep1
GSM8526278 C17_WAPLAID_C8,TACL-ON,WAPL,rep1
GSM8526279 C17_WAPLAID_C8,TACL-OFF,FLAG,rep1
GSM8526280 C17_WAPLAID_C8,TACL-OFF,FLAG,rep2
GSM8526281 C17_WAPLAID_C8,TACL-OFF,WAPL,rep1
GSM8526282 C17_WAPLAID_C8,TACL-ON_IAA,FLAG,rep1
GSM8526283 C17_WAPLAID_C8,TACL-ON_IAA,FLAG,rep2
GSM8526284 C17_WAPLAID_C8,TACL-OFF_IAA,FLAG,rep1
GSM8526285 C17_WAPLAID_C8,TACL-OFF_IAA,FLAG,rep2
GSM8545828 C17,cherry,rep1
GSM8545829 C17,cherry,rep2
GSM8545830 C17,TACL-OFF,rep1
GSM8545831 C17,TACL-OFF,rep2
GSM8545832 C17,TACL-ON,rep1
GSM8545833 C17,TACL-ON,rep2
GSM8545849 C17,TACL-ON,rep1, BrU-seq
GSM8545850 C17,TACL-ON,rep2, BrU-seq
GSM8545851 C17,TACL-ON,rep3, BrU-seq
GSM8545852 C17,TACL-OFF,rep1, BrU-seq
GSM8545853 C17,TACL-OFF,rep2, BrU-seq
GSM8545854 C17,TACL-OFF,rep3, BrU-seq

GSM8545855 C17,TmCherry,rep2, BrU-seq
GSM8545856 C17,TmCherry,rep3, BrU-seq
GSM8545857 C17,TmCherry_Dox2h,rep3, BrU-seq
GSM8559290 HAP1, C17, mCherry, rep1
GSM8559291 HAP1, C17, TACL-ON, rep1
GSM8559292 HAP1, C17, TACL-OFF, rep1
GSM8559293 HAP1, C17_CTCF-AID, TACL-ON, rep1
GSM8559294 HAP1, C17_CTCF-AID, TACL-ON, rep2
GSM8559295 HAP1, C17_CTCF-AID, TACL-ON_IAA, rep1
GSM8559296 HAP1, C17_PDS5A-AID, TACL-ON, rep1
GSM8559297 HAP1, C17_PDS5A-AID, TACL-ON_IAA, rep1
GSM8559298 HAP1, C17_RAD21-AID, TACL-ON, rep1
GSM8559299 HAP1, C17_RAD21-AID, TACL-ON_IAA, rep1
GSM8559300 HAP1, C17_STAG2-AID, TACL-ON, rep1
GSM8559301 HAP1, C17_STAG2-AID, TACL-ON_IAA, rep1
GSM8559302 HAP1, C17_WAPL-AID, TACL-ON, rep1
GSM8559303 HAP1, C17_WAPL-AID, TACL-ON_IAA, rep1
GSM8620453 C17,cherry
GSM8620454 C17,TACL-OFF
GSM8620455 C17,TACL-ON

Genome browser session (e.g. UCSC)

n/a

Methodology

Replicates

Most ChIP-seq experiments have been repeated at least twice.

Sequencing depth

All ChIP experiments were sequenced with at least 50bp single-end or paired-end reads. For ChIP experiments, 15 million reads were sequenced, and 5 million reads for input samples.

Antibodies

Anti-SMC1 (A300-055A, Bethyl), anti-SMC3 (A300-060A, Bethyl), anti-RAD21 (05-908, Merck), anti-NIPBL (A301-779A, Bethyl), anti-FLAG (F1804, Merck), anti-SCC4/MAU2 (ab183033, Abcam), anti-STAG1 (A302-579A, Bethyl), anti-STAG2 (A300-159A, Bethyl),anti-CTCF (ab128873, Abcam), anti-H3K4me3 (39060, Active motif), anti-H3K27ac (39133, Active motif), anti-V5 (R960-25, Thermo fisher), anti-WAPL (sc-365189, Santa Cruz), anti-PDS5A (A300-089A, Bethyl)

Peak calling parameters

ChIPseq peaks were filtered for a signalValue which represented clear peaks by visual inspection

Data quality

Data were routinely checked for quality control.

Software

Data were analyzed with 4DN pipeline.
