# Supplementary figures and images for: Characterization of induced cohesin loop extrusion trajectories in living cells
Source: Nat Genet. 2025 Oct 16;57(11):2785–97. doi: 10.1038/s41588-025-02358-0 (PMC12597828; doi:10.1038/s41588-025-02358-0)

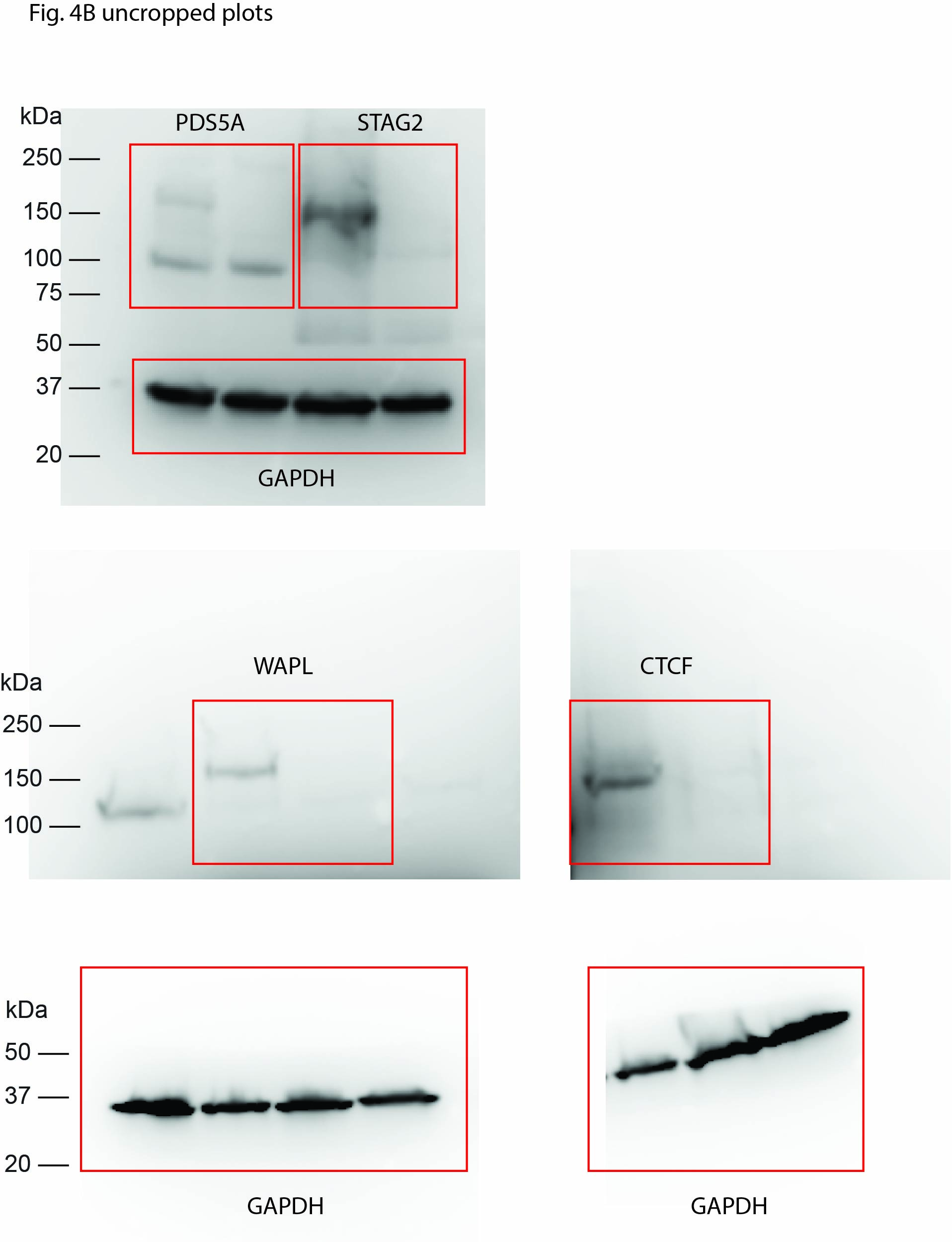

Supplement: Supplementary file 6 — Unprocessed western blots. [file 41588_2025_2358_MOESM6_ESM.jpg]

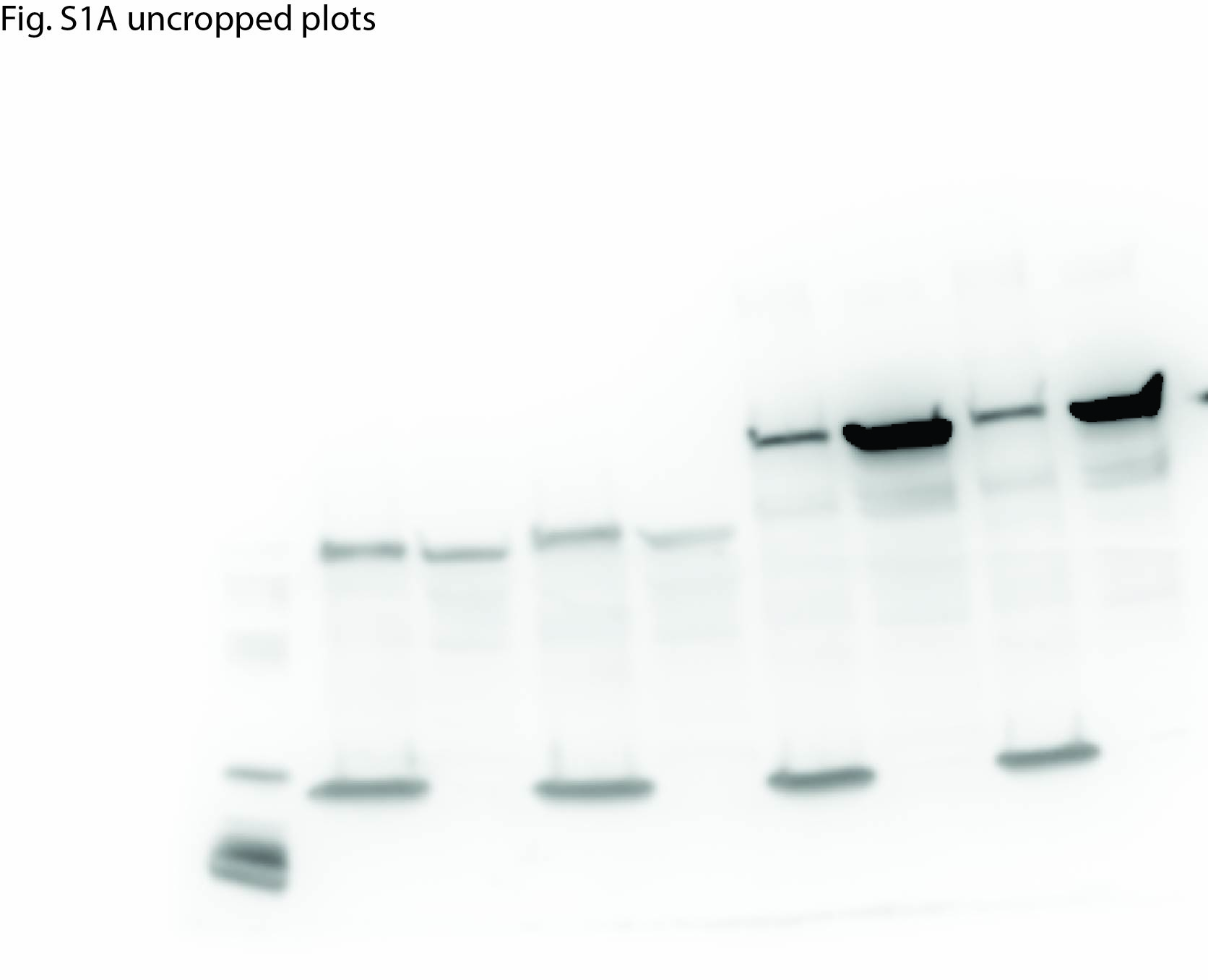

Supplement: Supplementary file 7 — Unprocessed western blots. [file 41588_2025_2358_MOESM7_ESM.jpg]

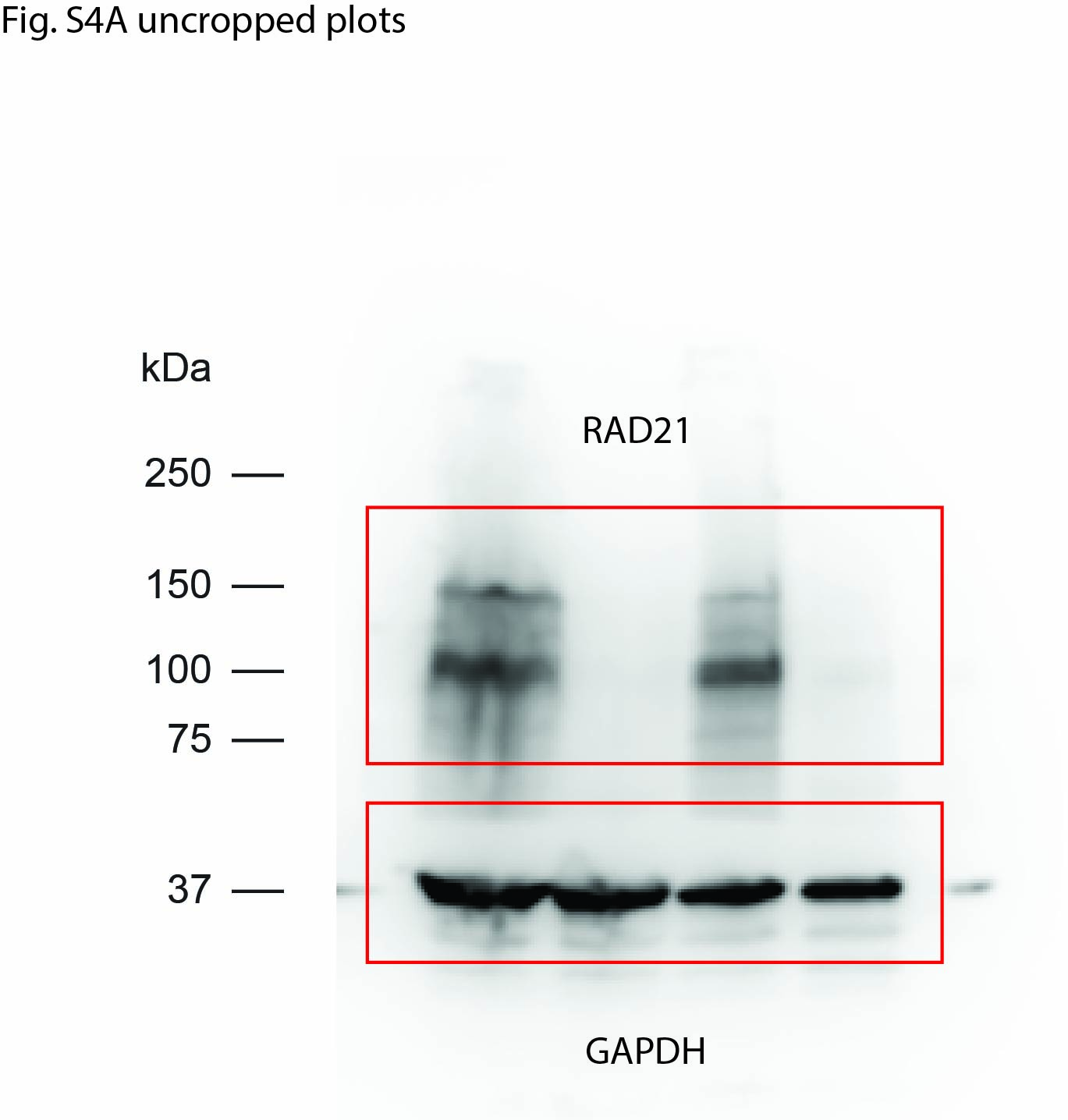

Supplement: Supplementary file 8 — Unprocessed western blots. [file 41588_2025_2358_MOESM8_ESM.jpg]

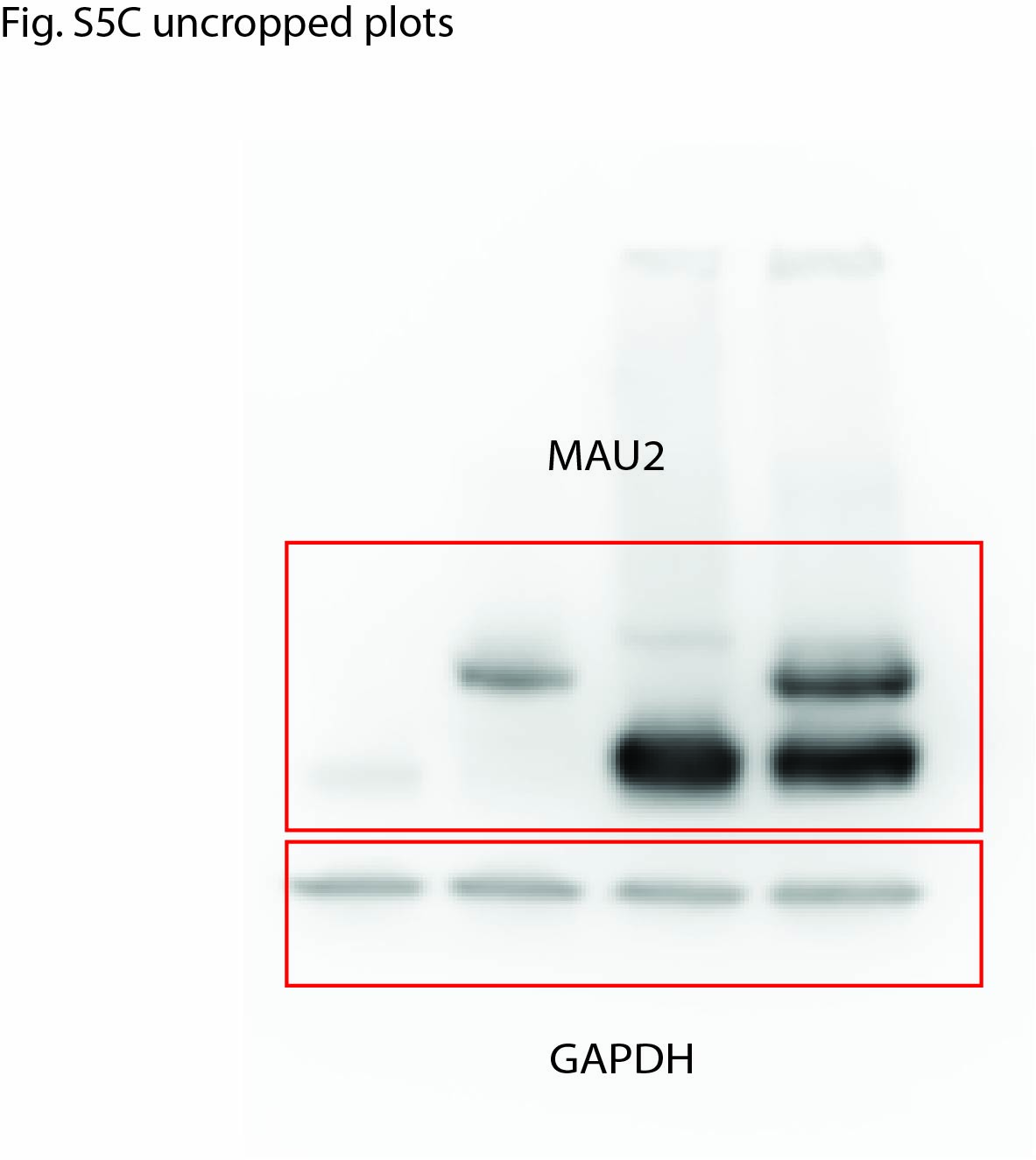

Supplement: Supplementary file 9 — Unprocessed western blots. [file 41588_2025_2358_MOESM9_ESM.jpg]
